# 3D-Printed Boron-Nitrogen Doped Carbon Electrodes for Sustainable Wastewater Treatment via MPECVD

**DOI:** 10.1007/s40820-025-01827-9

**Published:** 2025-06-24

**Authors:** Iwona Kaczmarzyk, Malgorzata Szopińska, Patryk Sokołowski, Simona Sabbatini, Gabriel Strugala, Jacek Ryl, Gianni Barucca, Per Falås, Robert Bogdanowicz, Mattia Pierpaoli

**Affiliations:** 1 Faculty of Electronics, Telecommunications and Informatics, Gdansk University of Technology, 11/12 Gabriela Narutowicza Street, 80-233 Gdańsk, Poland; 2 Faculty of Civil and Environmental Engineering, Gdansk University of Technology, 11/12 Gabriela Narutowicza Street, 80-233 Gdansk, Poland; 3 Department of Science and Engineering of Matter, Environment and Urban Planning (SIMAU) , Università Politecnica delle Marche, INSTM Research Unit, Via Brecce Bianche 12, 60131 Ancona, Italy; 4 Department of Materials Science and Technology, Institute of Manufacturing and Materials Technology, Faculty of Mechanical Engineering and Ship Technology, Gdańsk University of Technology, 11/12 Gabriela Narutowicza Street, 80-233 Gdansk, Poland; 5 Division of Electrochemistry and Surface Physical Chemistry, Faculty of Applied Physics and Mathematics, Gdańsk University of Technology, Narutowicza 11/12, 80-233 Gdansk, Poland; 6 Division of Chemical Engineering, Department of Process and Life Science Engineering, Lund University, PO Box 124, 221 00 Lund, Sweden

**Keywords:** Carbon nanowall, Phase inversion, Microwave plasma-enhanced chemical vapor deposition, Electrochemical oxidation, Additive manufacturing

## Abstract

This study proposes a novel and sustainable method for fabricating 3D-printed carbon-based electrodes for electrochemical wastewater treatment. We prepared B,N-doped carbon electrodes with hierarchical porosity and a significantly enhanced surface area-to-volume ratio (up to 180%) compared to non-optimized analogues using a synergistic combination of 3D printing, phase inversion, and microwave plasma-enhanced chemical vapor deposition. This process allows the metal-free growth of vertically aligned carbon nanostructures directly onto polymer-derived substrates, resulting in a 20-fold increase in the electrochemically active surface area. Computational fluid dynamics simulations were used to improve mass transport and reduce pressure drop. Electrochemical characterization demonstrated that the optimized electrodes performed significantly better, achieving 4.7-, 4-, and 6.5-fold increases in the degradation rates of atenolol, metoprolol, and propranolol, respectively, during electrochemical oxidation. These results highlight the efficacy of the integrated fabrication and simulation approach in producing high-performance electrodes for sustainable wastewater treatment applications.

**Supplementary Information:**

The online version contains supplementary material available at 10.1007/s40820-025-01827-9.

## Highlights

- A novel approach combining 3D printing, phase inversion, and microwave plasma-enhanced chemical vapor deposition is presented. This technique enables the creation of carbon-based electrodes with precise micro- and nanoscale control, offering potential for sustainable and high-performance wastewater treatment applications.
- The synthesized 3D carbon scaffolds, enriched with B,N-doped carbon nanostructures, demonstrated superior performance in the electrochemical oxidation of β-blockers. Computational fluid dynamics simulations were used to optimize electrode design, leading to improved mass transport and reaction kinetics.
- This research provides a sustainable and scalable solution for removing emerging contaminants from wastewater. The catalyst-free approach simplifies the fabrication process and reduces potential material contamination, making it a promising technology for advanced water treatment applications.

**Supplementary Information:**

The online version contains supplementary material available at 10.1007/s40820-025-01827-9.

## Graphical abstract

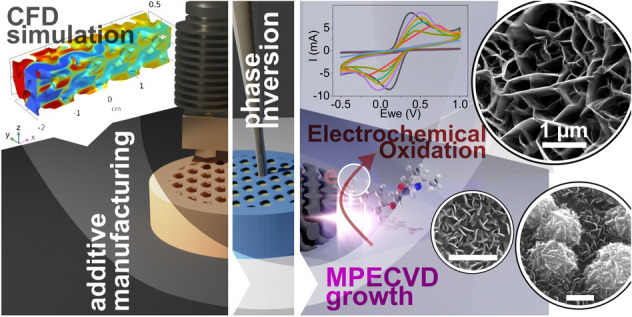

## Introduction

Three-dimensional micro-/nanofabrication is a powerful technology for precision applications. While additive manufacturing is cost-effective and widely accessible, it often lacks the necessary nanoscale precision and is restricted by the limited range of compatible thermoplastic polymers. Template-assisted fabrication, which employs 3D-printed polymer scaffolds as molds, provides a novel approach to increasing the range of usable materials [1, 2], enabling customized architectures for energy storage and sensing devices, offering control over electrode geometry, mass loading, and porosity [3, 4]. However, this method can encounter difficulties in controlling surface porosity, which can have a significant impact on the development of materials for advanced electrochemical applications. In these cases, precise porosity control is crucial for efficient mass transport and reaction performance. The design of sustainable, carbon-based electrodes with hierarchical porosity poses a considerable challenge due to the necessity of compatibility at both the atomic and macroscopic levels. Additionally, achieving different levels of porosity in a controlled manner is challenging [5]. The combination of additive manufacturing and topology optimization presents a viable solution, enabling the fabrication of complex geometries beyond the capabilities of conventional manufacturing while allowing for rapid production and design flexibility. In this regard, computational fluid dynamics (CFD) simulations also play a pivotal role in optimizing flow patterns to ensure efficient reactant distribution and product removal in electrochemical cells [6]. These simulations can guide the design of electrode structures such as microarchitected flow-through electrodes, thereby enhancing power efficiency in energy storage systems [7]. Triply periodic minimal surfaces (TPMS) are repeating structures with zero mean curvature, high surface area-to-volume ratio, and found a place in catalysis-related and heat exchangers applications [8–10]. Indeed, in the context of electrode design, TPMS offers a way to create highly porous and optimized structures that enhance mass transfer performance by providing large surface areas for reaction sites and efficient mass transport. However, while TPMS architectures offer excellent theoretical advantages, their practical implementation in electrochemical devices requires materials that can be precisely structured at the micro- and nanoscale. Phase inversion-assisted molding of polyacrylonitrile (PAN) presents a viable approach, allowing the formation of porous, electrically conductive scaffolds that retain the benefits of TPMS structures after carbonization. The phase inversion method, a widely employed technique for creating porous polymeric structures, involves the dissolution of a polymer in a solvent, the casting of the resultant film, and subsequent immersion in a non-solvent bath. The rate of solvent exchange during this process dictates the porosity of the final structure [11, 12]. PAN is frequently utilized in this process due to its favorable chemical resistance properties and its capacity for straightforward carbonization into nanostructured carbon materials [13]. While many thermoplastic polymers are unsuitable for high-temperature applications or lack the necessary mechanical stability, PAN enables the fabrication of robust, conductive carbon scaffolds, thus overcoming material limitations in additive manufacturing.

Recent studies have focused on growing one-dimensional carbon nanotubes (CNTs) on carbon fibers [14, 15] or incorporation of metal nanoparticles [16] to enhance electrochemical performance. However, these studies are mostly limited to 1D structures. Expanding this work to 2D nanostructures, such as carbon nanowalls (CNWs), opens up new possibilities. Our group, and other research teams, have demonstrated that coating carbonized PAN fibers with CNWs during microwave plasma-enhanced chemical vapor deposition (MPECVD) enhances their crystallinity and electrochemical properties [17, 18], since pyrolysis offers practical advantages including procedural simplicity, scalability, and the ability to generate abundant defects and anchoring sites for metal catalysts [19]. Moreover, PAN fibers are inherently limited to 1D shapes, necessitating the development of novel methodologies for the fabrication of 3D structures from PAN. A promising approach is the integration of 3D printing with wet spinning, a technique that has the potential to generate more intricate geometries and to accommodate a broader spectrum of polymers. However, the approach is still limited by the availability of suitable materials. The application of topologically optimized 3D-printed structures for wastewater treatment is also an area that's not yet fully explored. The EU's new urban wastewater directive, which requires the removal of organic micropollutants from wastewater, highlights the need for innovative treatment technologies [20]. Electrochemical oxidation has been identified as a promising solution, given its ability to remove a wide range of organic pollutants and the potential to replace conventional methods such as ozone treatment and activated carbon [21–23]. However, the majority of electrodes utilized in this process are dependent on critical raw materials and are operated in a flow-by configuration, which restricts their effectiveness [24, 25]. The adoption of carbon-based electrodes in conjunction with a flow-through configuration has been demonstrated to enhance efficiency by extending the reaction time, thereby facilitating the removal of organic pollutants [26].

To overcome these challenges, this study presents a novel approach for synthesizing 3D carbon scaffolds enriched with B,N-doped carbon nanostructures, optimized for advanced electrochemical oxidation of pharmaceuticals. The performance of the fabricated electrodes was evaluated in the electrochemical oxidation of three β-blockers: propranolol, atenolol, and metoprolol. Transformation product analysis using UHPLC/MS allowed for the identification of degradation pathways on different electrode architectures, providing valuable insights into the effectiveness and mechanisms of pollutant removal. CFD was used to investigate 15 different modular blocks, including TPMS and 3D fractal geometries, with the 5 most performing designs selected based on their high surface area-to-volume ratio, enhanced mixing capabilities, and reduced pressure drop, for efficient electrochemical oxidation. Various carbon nanostructures were then grown directly on the PAN-carbon scaffold without the use of any catalysts. A Taguchi design was employed to identify the optimal conditions for MPECVD growth, ensuring the best electrochemical performance of the electrode. This catalyst-free approach simplifies the fabrication process and reduces potential material contamination. This study presents a comprehensive approach, from optimal material design to synthesis, and demonstrates how this innovative combination of techniques enables the creation of highly efficient and sustainable electrodes for advanced water treatment applications.

## Experimental Section

### Scaffold Design and CFD Simulation

The unique advantage offered by TPMS geometries in electrochemical applications is attributable to their interconnected porous structures, which improve reactant diffusion while minimizing pressure drop. This makes them particularly suitable for electrochemical water treatment, where efficient mass transfer is critical for high degradation rates. In the present study, CFD simulations were conducted under laminar flow conditions and steady-state operation, which are representative of practical electrochemical environments. The fluid was modeled as an incompressible Newtonian liquid to simulate the behavior of wastewater. The simulations evaluated mixing efficiency, pressure drop, and the surface area-to-volume (A/V) ratio, which are all essential parameters for the optimization of electrochemical reactions. As outlined in Table S1, the implicit equations defining the TPMS structures in this study were adapted to ensure compatibility with 3D printing by incorporating surface thickening. The models were generated using MSLattice software [27], and to create the simulation domain, the stereolithography (STL) geometries were cleaned of potential self-intersections and any errors in the original file prior to numerical simulation. The COMSOL Multiphysics software was utilized to simulate fluid flow and mass transfer in seven periodic structures. In order to simplify the simulation, a small section (1 × 1 × 4-unit cells) of the structure was modeled and periodic boundary conditions were applied. This approach assumes that the structure is large enough for the effects of the outer boundaries to be negligible. The laminar flow solver of COMSOL was utilized to ascertain the hydraulic properties, with periodic boundary conditions employed on opposing faces perpendicular to the initial flow direction. Simulations were conducted at varying Reynolds numbers (Re), with the hydraulic diameter defined as follows:

1 $${d}_{h}=\frac{4V}{{A}_{\text{s}}}$$

where *V* is the volume of the fluid domain (cm^3^) and *A*_s_ the wetted surface area of the structure (cm^2^). The resistance to flow in the structures is quantified using the friction factor (*f*):

2 $$f = \frac{\tau }{{0.5 \cdot \rho .v^{2} }}$$

where *τ* is the wall shear stress (Pa), *ρ* is the fluid density (kg m^−3^), and *v* is the average velocity (m s^−1^). To calculate the wall shear stress, a force balance is applied to the fluid domain, resulting in the following relationship:

3 $$\tau \cdot A_{{\text{s}}} = \Delta P \cdot A_{{\text{c}}}$$

where Δ*P* is the pressure difference across the structure (Pa) and *A*_c_ the cross-sectional area of the inlet (m^2^). The coefficient of variation (CoV) is a metric that quantifies the relative local concentrations at a given cross section in relation to the mean. In the context of static mixers, it serves as an indicator of the mixing efficiency of the device, with a lower CoV signifying a more uniform concentration distribution and thereby reflecting enhanced mixing performance.

### Scaffold Fabrication

The internal topology of the mold determines the final electrode shape. Molds were fabricated using a fused deposition modeling (FDM) 3D printer (Creality Ender 3) with various commercially available, water-soluble filaments (1.75 mm diameter). Two of these filaments were identified as polyvinyl alcohol (PVA) and butenediol vinyl alcohol copolymer (BVOH)-based, while the composition of the remaining three filaments was not disclosed. The commercial names and printing parameters were followed according to the manufacturer's recommendations for each filament, which are reported in the supplementary materials (Table S2). Polyacrylonitrile (PAN) (MW 150,000, purity > 99%) was sourced from AmBeed, and dimethylformamide (DMF, purity > 99%) was purchased from Eurochem BGD. Molding and phase inversion were carried out by casting a 10% w/w PAN/DMF solution into the 3D-printed mold, which was then placed on a poly(methyl methacrylate) (PMMA) support within a stirred beaker of deionized water. This process lasted for 24 h, with one water change to ensure complete mold dissolution and solvent diffusion.

### Simultaneous Scaffold Pyrolysis and Nanostructure Growth

The process involved simultaneous pyrolysis, surface etching and secondary nanostructure growth, with the use of a MPECVD system (SEKI Technotron AX5400S, Japan). The experimental process was conducted for a duration of 40 min, with the initial 5 min dedicated exclusively to the generation of H_2_ plasma at a flow rate of 300 ccm, with the purpose of etching the amorphous carbon layer. The process was conducted under specific conditions, namely microwave power (600, 700, 800 W), total pressure (20, 30, 40 Torr), temperature (550, 600, 650 °C), and gas composition (0.075, 0.1, 0.125 CH_4_:H_2_). To optimize the number of tests, the experimental design followed the Taguchi L9 scheme (Table S3) on the PVA-molded scaffolds. MPECVD growth was performed in the presence of B_2_H_6_ as a boron precursor gas without seeding. Boron doping enhances electrical conductivity by introducing p-type carriers, thereby facilitating improved electron transfer during reactions [28]. It has also been demonstrated that boron doping increases active sites through defective structures, thereby boosting pollutant degradation efficiency [29]. Additionally, boron doping has been shown to positively influence carbon nanostructure growth, such as in BCNWs, promoting vertical graphene stacks that enhance surface area and facilitate faster electron transfer [30].

### Morphological, Chemical, and Electrochemical Characterization

The surface morphology of the electrodes was investigated using a Quanta FEG 250 (FEI) Schottky field-emission scanning electron microscope (SEM) equipped with a secondary electron (ET) detector with a beam accelerating voltage of 15, 17.5, and 20 kV. Transmission electron microscopy (TEM) techniques were used to investigate the carbon nanostructures on the electrodes. The analysis was carried out with a Philips CM200 electron microscope equipped with a LaB6 filament, operating at 200 kV. For TEM observations, a portion of the electrode surface was gently scratched with a lancet, and the resulting powder was dispersed in ethanol and sonicated for approximately 1 min. A drop of the suspension was then deposited onto a commercial carbon-holed TEM grid and allowed to air-dry. Surface chemical analysis was performed using X-ray photoelectron spectroscopy (XPS) with a ThermoFisher Scientific Escalab 250Xi multispectroscope, equipped with an Al Kα X-ray source. Measurements were taken with a 650 μm spot size and a pass energy of 20 eV. To neutralize surface charge buildup, a combination of low-energy electrons and argon ions was employed. The collected spectra were calibrated using the C 1*s* peak at 284.6 eV. Computed microtomography (µCT) was employed for the three-dimensional reconstruction of the actual geometry of the samples. The imaging was performed using a GE phoenix v|tome|xs system, with the X-ray power configured at 30 W (100 kV, 300 μA) and an exposure time of 5000 ms per radiogram. The resolution of the 3D reconstructions was 29.99 μm per voxel. Model alignment and comparison were performed using CloudCompare [31]. Raman spectroscopy was conducted using a Horiba Jobin Yvon LabRam Aramis Raman spectrometer with a 632.8-nm laser. The system incorporated an Olympus BX41 confocal microscope for precise sample focusing and a Synapse CCD camera for detection. The IR spectra were recorded using a Bruker Invenio-R spectrometer equipped with either a Ge ATR crystal or a Harrik Praying Mantis diffuse reflection accessory. All DRIFTS spectra were measured with 256 scans and at a resolution of 4 cm^−1^, while ATR spectra were measured with 128 scans. All voltammetric and impedance measurements were performed using a VMP-300 potentiostat–galvanostat (Bio-Logic, France) controlled by EC-Lab software. The experimental setup comprised a three-electrode configuration, with Ag/AgCl/3 M KCl serving as the reference electrode and a platinum wire designated as the counter electrode. Cyclic voltammetry (CV) measurements were conducted in a 2.5 mM K_3_[Fe(CN)_6_] + 2.5 mM K_4_[Fe(CN)_6_] solution in 0.05 M PS at varying scan rates: 5, 10, 25, 50, 100, and 200 mV s^−1^. Electrochemical impedance spectroscopy (EIS) experiments were recorded at open circuit potential across a frequency range of 0.1 Hz to 200 kHz with a sinusoidal amplitude of 10 mV.

### Electrochemical Oxidation Test and UHPLC Analysis

Analytical standards, isotopically labeled standards, and deuterated standards were procured from Sigma-Aldrich (Merck, Germany). Eluent additives, including ammonium formate and formic acid, suitable for mass spectrometry (LiChropur™, Merck, Germany), and hypergrade acetonitrile (ACN) for LC–MS (LiChrosolv®, Merck, Germany) were used. MS-grade water (LiChrosolv®, Merck, Germany) was used for stock solution and eluent preparation, while ultrapure water (resistivity 18.2 MΩ cm at 25 °C) was obtained from a Direct-Q® Water Purification System and used for the cleaning of laboratory glassware. Polytetrafluoroethylene (PTFE-20/15 MS) 0.2-µm-pore-size syringe filters were obtained from Chromafil® (Machery-Nagel, Germany). Solvent evaporation was performed under a nitrogen stream using a Turbovap LV (Biotage, Sweden) equipped with an N_2_ generator (Generator Genius XE35 230 V, PEAK Scientific, Great Britain). A 50-mL sample, prepared at a 1:50 dilution, was then analyzed. Solid-phase extraction (SPE) was employed as a sample preparation step, without prior filtration. Hydrophilic–lipophilic balanced (HLB) 500-mg SPE cartridges (Oasis, Waters) were used, and the SPE procedure is detailed in the supplementary material (S1). One milliliter of the reconstituted (MeOH) sample was filtered (Chromafil PTFE-20/15 MS, 0.2-µm-pore-size syringe filter) prior to UHPLC-ESI–MS/MS analysis. An ultra-high-performance liquid chromatography tandem mass spectrometry system with electrospray ionization (UHPLC-ESI–MS/MS; Nexera XR coupled with LC/MS-8050, Shimadzu) was used to determine the β-blockers metoprolol tartrate (Met), atenolol (Ate), and propranolol hydrochloride (Pro), all pharmaceutical secondary standards, certified reference material, as well as their degradation products. The analysis of Met, Ate, and Pro was conducted using multiple reaction monitoring (MRM), while the evaluation of degradation products was undertaken using single ion monitoring (SIM). Relative recoveries were 77.13%, 71.4%, and 76.8% for Met, Ate, and Pro, respectively. A full description of the UHPLC-ESI–MS/MS method is provided in Supplementary Material.

## Results and Discussion

### Topological Optimization of the Scaffold by CFD Simulation

Considering the manufacturing limitations, a total of 15 building blocks, among which 6 TPMS and 2 fractal geometries, were selected (Table 1). Each TPMS was fabricated at different densities (*δ*), representing the ratio between the effective solid volume and the total occupied one. For fractal geometries, *δ* corresponds to the number of iterations which were performed.

**Table 1:** Geometries tested in CFD simulation

Type	Primitive			*δ* range	*f*_0.5_ [–]	*A*_s_ [cm^2^]	*A*_s_/*V* [cm^2^ cm^−3^]
TPMS	Diamond	Sheet	dia^+^	0.2–0.6	31.8Re^−0.78^	26.9	6.7
		Solid	dia^−^	0.2–0.6	45.5Re^−0.9^	15.2	3.8
	Fischer–Koch S	Solid	fks^−^	0.3–0.6	23.1Re^−0.71^	21.5	5.4
	Gyroid	Sheet	gyr^+^	0.3–0.6	15.1Re^−0.81^	21.7	5.4
		Solid	gyr^−^	0.3–0.6	28.2Re^−0.72^	12.4	3.1
	Schoen's I-WP	Sheet	iwp^+^	0.4–0.7	17.1Re^−0.88^	24.8	6.2
		Solid	iwp^−^	0.3–0.7	15.0Re^−0.78^	14.3	3.6
	Neovius	Sheet	neo	0.3–0.6	10.5Re^−0.62^	14.7	3.7
		Solid	neo^−^	0.3–0.6	176Re^−0.55^	14.1	3.5
	Schwarz P-Surface	Sheet	pri^+^	0.3–0.6	23.5Re^−0.87^	16.1	4.0
		Solid	pri^−^	0.3–0.6	72.5Re^−0.61^	9.4	2.4
Fractal	Menger sponge	Sheet	men^+^	2–3*			
		Solid	men^−^	2–3*			
	Koch quadratic	Sheet	koc^+^	2–3*			
		Solid	koc^−^	2–3*			

^*^For fractal geometries, *δ* is the fractal iteration number

**Fig. 1:**
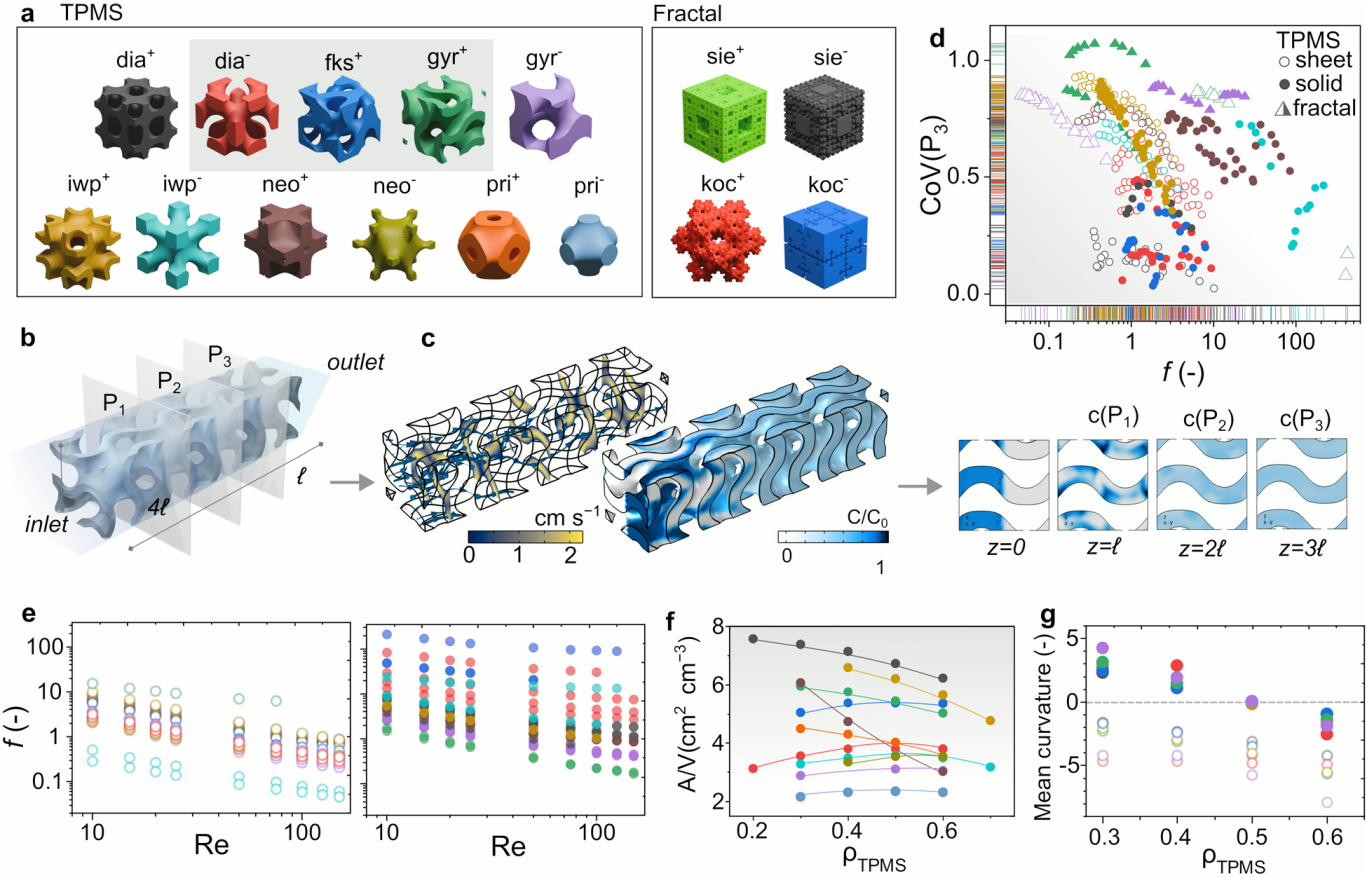
Results of the CFD simulation. **a** Evaluated TPMS and fractal 1 × 1 × 1 cells. **b** Computational domain with the three planes at which the CoV was evaluated. **c** Velocity and normalized concentration distribution plot within the solid domain. **d** Plot of the CoV(P_3_) against f for the evaluated geometries and **e** its dependence on Re. **f** Plot of the wet surface/volume ratio and **g** mean curvature against the TPMS thickness. The symbol colors of **d**–**g** refer to the TPMS color in **a**

A 4 × 1 × 1 cm model was created for each selected triply periodic minimal surface (TPMS) and fractal geometry (Fig. 1a), representing the actual volume occupied by the fluid. An example of gyr^+^ with *δ* = 0.4 is shown in Fig. 1b. Following numerical simulation, both hydraulic properties and mixing efficiency were evaluated (Fig. 1c).

Figure 1d displays the relationship between the friction factor and the coefficient of variation (CoV) calculated at a distance equal of 3ℓ from the inlet for all CFD simulations. Geometries located in the lower left quadrant of the plot are preferable, as they demonstrate both reduced resistance to fluid flow against the model surface and enhanced mixing. It is noteworthy that fractal-based models (triangles) demonstrate poor mixing (and among the lowest *f*) or the highest mixing (lowest CoV) with the highest value of *f*. Figure 1f presents the area- to-volume (A/V) ratio for each geometry at different values of *δ*. Based on these results, dia^−^, fks^−^, and gyr^+^ were selected, along with dia^+^ and pri^−^, due to the highest and lowest surface/volume ratios, respectively. For solid networks, the maximum surface/volume ratio occurs at *δ* = 0.5, whereas for sheet network-based TPMS, this ratio decreases monotonically with increasing density. The correlation between friction coefficient (*f*) and Reynolds number (Re) for both TPMS parts, as depicted in Fig. 1e, illustrate that "sheet" geometries exhibit the lowest friction. Moreover, the thickening of the TPMS surface results in a nonzero mean curvature, but mean curvature approaches zero in "sheet" geometries with lower thickness or in "solid" counterparts with 50% void fraction (Fig. 1g).

### Scaffold Synthesis: Phase Inversion-Assisted Micromolding

**Fig. 2:**
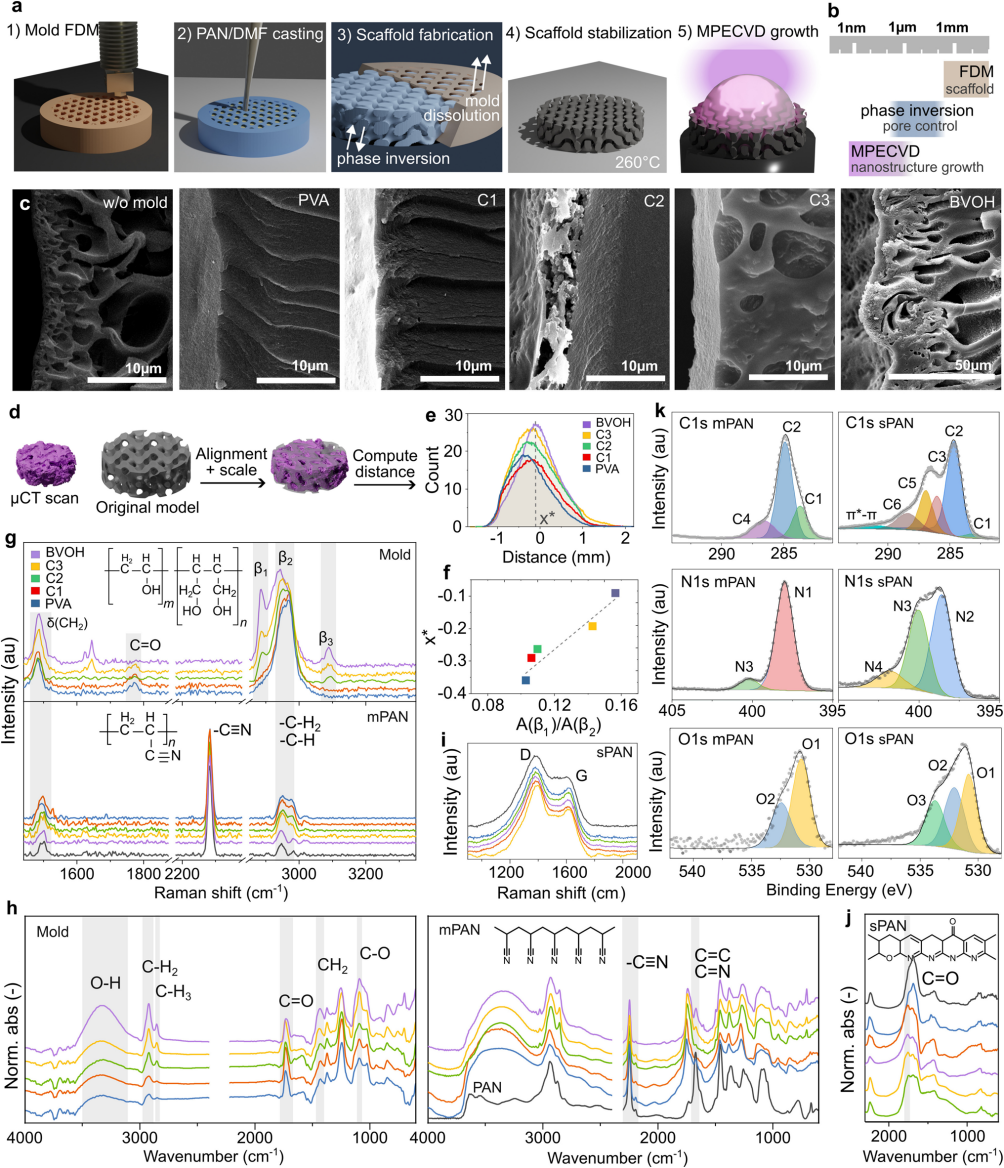
Carbon scaffold molding. **a** Schematic representation of the 3D printing molding process and **b** of the electrode structure control. **c** SEM micrographs of the cross section of the PAN casted in different molds and the **d** procedure to assess their similarity to the digital model, with **e** the calculated distances distribution and **f** their maximum position against the Raman peaks ratio. **g** Raman and **h** FTIR spectra of the 3D-printed molds, the resulting molded PAN and **i, j** after stabilization. **k** High-resolution C1*s*, N1*s* and O 1*s* XPS spectra of molded PAN (mPAN) and stabilized PAN (sPAN).

Molds were fabricated using fused deposition modeling (FDM) with various commercially available filaments and subsequently employed for scaffold casting, as illustrated in Fig. 2a. The strategy employed to control the hierarchical porosity is illustrated in Fig. 2b.

The 3D printing settings were based on the recommendations provided by the filament manufacturers. Following fabrication, the molds were immersed in water in order to facilitate mold dissolution and solvent transfer from the solution to the water bath. Polyacrylonitrile (PAN) consists of linear chains of repeating acrylonitrile units, and the stabilization process transforms it into a ladder polymer with conjugated double bonds and cross-linked networks. Subsequent pyrolysis promotes the formation of graphitic domains. Traces of aluminum (Al) were detected by energy-dispersive X-ray spectroscopy (EDX) in all the molded samples (Table S4). The varying filament compositions influenced the phase inversion process, resulting in diverse porosity types and dimensions (Fig. 2c). Especially, larger pores were formed by the BVOH mold compared to the PVA mold. To examine the resulting geometry and porosity, µCT was employed. For each sample, both the 3D images and the corresponding 2D cross sections are presented in Fig. S1. Voids and pores are highlighted to enable a clear comparison of porosity and structural variations between scaffolds. Figure 2d presents a representative reconstructed µCT model (BVOH), with the original model shown on the right. To compare the impact of mold composition, µCT-reconstructed models were aligned and scaled to the original model using CloudCompare software. The distance between the real and digital models was calculated, and the distribution height was normalized to the total volume estimated by µCT and is presented in Fig. 2e. Interestingly, the distributions are asymmetrical, with the peak shifting toward zero as the BVOH content—expressed as *A*(*β*_1_)/*A*(*β*_2_)—increases (Fig. 2f). This suggests that a higher butenediol concentration in the vinyl alcohol mixture minimizes deformation in the molded sample. Volumetric shrinkage analysis revealed that pure BVOH exhibited the highest shrinkage (82.4%), while the other samples showed comparable shrinkage, averaging 72.5% (SD 1.5%). Table 2 summarizes the computed micro-tomographies (µCT) of the synthesized electrodes. The results of these investigations showed that the C1 sample had the highest total porosity at 80%. The total porosity of the PVA, C2, and C3 samples ranged between 79% and 72%. The lowest total porosity was observed for the BVOH sample, which was slightly less than 62%. A pore analysis was also carried out, showing that the four samples had pore contents ranging from 0.3 to 0.4%. The BVOH sample, which had the lowest total porosity, had an increased pore content of 0.8%. Solvent exchange rate tests were conducted by monitoring the PVA/water ratio over time, measuring the dissolution of 1% PVA/water and 1% BVOH/water solutions via FTIR. The CH_2_-related peak at 2980 cm^−1^ was selected for the polymer, while the water-specific peak at 1635 cm^−1^ was chosen over the broader O–H peak at 3370 cm^−1^. Results indicate that PVA and BVOH dissolution begins after approximately two hours, during which polymer solvation and potential chain relaxation enhance peak intensities at 2890 and 2980 cm^−1^. No significant differences were observed between BVOH and PVA (Fig. S2). Thus, the increased porosity was attributed to the butadiene units in the BVOH mold, which increased hydrophobicity and compatibility with the PAN solvent, DMF. This led to simultaneous rather than sequential solvent exchanges, promoting the formation of a more porous and irregular surface (Fig. 2c). Also, the addition of cosolvents was investigated, with a 20% acetone solution promoting diffuse micron-scale porosity near the surface by increasing diffusion rates, which accelerates local polymer precipitation and leads to the formation of smaller, more uniformly distributed micropores (Fig. S3).

**Table 2:** Calculation of porosity for tested samples based on 3D reconstructions from computed microtomography

_Sample_	_ROI Volume_	_ROI solid volume_	_ROI voids volume_	_ROI pores volume_	_Void in the sample_	_Pore in the sample_	_Total porosity_	_Volumetric shrinkage_
	(mm^3^)	(mm^3^)	(mm^3^)	(mm^3^)	(%)	(%)	(%)	(%)
_PVA_	_2732_	_571_	_2153_	_8.7_	_78.8_	_0.3_	_79.1_	_71.9_
_C1_	_2460_	_491_	_1961_	_7.9_	_79.7_	_0.3_	_80.0_	_71.7_
_C2_	_1868_	_524_	_1337_	_7.7_	_71.6_	_0.4_	_72.0_	_74.4_
_C3_	_2113_	_470_	_1636_	_7.3_	_77.4_	_0.3_	_77.8_	_71.5_
_BVOH_	_1531_	_587_	_931_	_12.4_	_60.8_	_0.8_	_61.7_	_82.5_

FTIR and Raman analyses were used to investigate the composition of the filaments. While the spectral profiles of the 5 samples are similar, they appear to be composed of polyvinyl alcohol (PVA) and butenediol vinyl alcohol (BVOH) in varying ratios. In the Raman spectra of the mold (Fig. 2g, top), the most intense band, centered at 2945 cm^−1^, is attributed to –CH_2_ stretching vibrations. Interestingly, two peaks at [*β*_1_] and [*β*_2_] seem to be related to the butenediol-containing samples. These peaks likely correspond to the C–H stretching vibrations of methylene (CH_2_) groups [32] in the polymer chain, suggesting a correlation with butenediol concentration. FTIR spectra (Fig. 2h, left) further confirm the mold composition. Despite the similarity in the spectral profiles of the 5 samples, the presence of both PVA and BVOH is evident. A significant band at 3345 cm^−1^ is related to OH groups, while intense bands at 2921 and 2848 cm^−1^ are attributed to CH_2_ and CH stretching, respectively. The band at 1094 cm^−1^ is associated with C–O stretching vibrations, which can be related to the degree of PVA crystallinity [33]. After casting polyacrylonitrile (PAN) by dissolving the mold and subsequently removing the solvent, the resulting Raman spectra (Fig. 2g, bottom) exhibit minimal variation among the samples, aside from the appearance of the strong C≡N peak. The Fourier transform infrared spectra (Fig. 2h, right) consistently highlight the characteristic PAN absorption bands at 2244, 1668, 1453, 1360, and 1251 cm^−1^. These bands exhibit slight shifts, likely attributable to chemical interactions between PAN and the polyvinyl alcohol (PVA)/butenediol vinyl alcohol (BVOH) mold. Additionally, the presence of a carbonyl band at 1734 cm^−1^ provides further evidence of PVA and BVOH residues from the mold.

Upon stabilization, only the D and G bands remain observable in the Raman spectra (Fig. 2i), signifying the formation of a ladder-like thermoset structure, irrespective of the mold used. This stabilization process involves the reaction of nitrile (C≡N) groups in PAN to form cyclic structures or their interaction with oxygen, leading to the incorporation of oxygen-containing functional groups such as carbonyl (C=O) and hydroxyl (–OH) groups. Figure 2j shows the post-stabilization spectra, highlighting molecular alterations, particularly within the hydrocarbon chains. Despite these changes, the characteristic PAN triple bond remains intact and loses intensity. The emergence of new unsaturated bonds is evident from the bands observed between 1600 and 1800 cm^−1^, while the original structures of PVA and BVOH are absent. The removal of hydrogen atoms facilitates the formation of unsaturated bonds and enhances conjugation, as also depicted in Fig. 2k.

Considering the PAN after molding, stabilization, and carbonization, the C1*s* spectrum was fitted into seven peaks, according to other authors [34, 35]: C=C (C1), C–C (C2), C–O/C = N (C3), C≡N (C4), C=O (C5), COOR (C6), and π*–π* at binding energies of 283.3, 284.8, 285.9, 286.6, 287.0, 288.4, and 290.9 eV, respectively. In molded PAN, the C 1*s* spectrum shows peaks for C1, C2, and –C≡N, while the N1s spectrum should be dominated by a single peak (N1) at ~ 398 eV (C≡N) [34]. A minor peak (N2) at 400.2 eV appears, related to the –C–N or pyrrolic nitrogen [36]. After oxidation in air, the C4 peak disappears, indicating the complete cyclization of C≡N. As nitrile groups cyclize to form conjugated structures, particularly pyridine-like rings (C = N), a new peak emerges at ~ 286 eV [37]. At the same time, C5 and C6 increases evidently, which reveals strong oxidation reactions at this stage. The nitrile N1*s* peak (~ 398.8 eV) diminishes with cyclization, giving rise to a new peak at around 400 eV (pyridonic + pyrrolic) [13]. A higher binding energy component (~ 401.5 eV) also appears, attributed to oxidized nitrogen (N–O or N–C=O) formed during oxidative stabilization. Intrinsic nitrogen incorporation alters the electronic structure of the carbon framework, introducing electron-rich regions that enhance charge transfer properties. Pyridinic and graphitic nitrogen functionalities have been reported to facilitate redox reactions, improving the catalytic activity of carbon-based electrodes [38].

### MPECVD-Assisted Nanostructure Growth

**Fig. 3:**
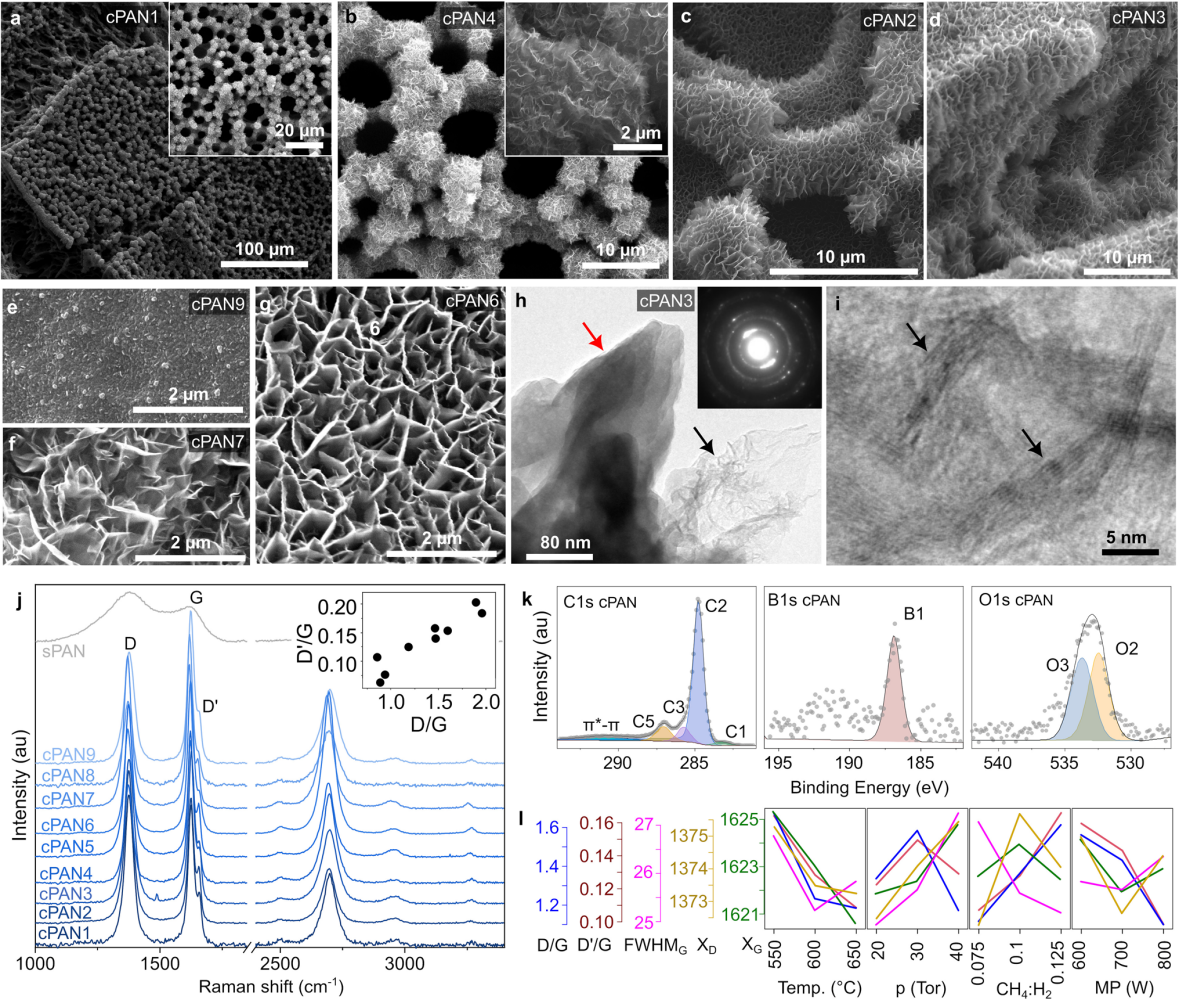
Characterization of the MPECVD-overgrown nanostructures. **a–g** SEM of cPAN, **h** bright field TEM image of carbonaceous nanostructures having bulk (red arrow) or thin (dark arrow) nanowall-like structures and the corresponding SAED pattern (inset). **i** HR-TEM image of folded thin nanowall-like structures showing lattice planes of graphite (dark arrows).** j** Raman spectra after stabilization. **k** High-resolution XPS spectra in C 1*s*, B 1*s*, and O 1*s* binding energy range, with proposed peak deconvolution model and **l** effect plot

Growth conditions, detailed in the supplementary materials, were optimized using an L9 Taguchi orthogonal array. This approach allowed for efficient investigation of the effects of four factors: temperature, pressure, microwave power, and gas composition, each at three different levels. From SEM analysis, after conducting simultaneous pyrolysis and CVD growth, different types of carbon nanostructures were formed on both flat surfaces and in open porosity due to the simultaneous plasma etching of the outer carbon layer (Fig. 3a–g). It has been reported that the CVD process can significantly improve the material's electrical conductivity due to the growth of CNTs, fiber-like structures [29], or two-dimensional nanostructures [18], and the transformation of amorphous carbon into graphitized carbon [14]. In this study, we observed primarily the formation of graphitic sheets oriented perpendicular to the surface, known also as carbon nanowalls [39, 40].

In samples exhibiting well-developed carbon nanowalls (cPAN2, cPAN3, and cPAN5), these structures were found to be uniformly distributed across both flat surfaces and rough open porosity. This observation suggests that specific conditions were conducive to the uniform growth of nanowalls across diverse surface morphologies. Conversely, samples cPAN1, cPAN4, cPAN6, and cPAN7 exhibited a diversity of nanostructures, comprising poorly developed BCNWs, petal-like structures, and sphere-like agglomerates, which appear to be contingent on local conditions such as plasma exposure and temperature gradients (Fig. S4). For instance, sample cPAN1 exhibited a combination of poorly developed BCNWs and petal-like nanostructures, suggesting insufficient B-radical availability or reduced temperatures impeding optimal nanowall renucleation. In a similar manner, samples cPAN8 and cPAN9 displayed very poorly developed nanostructures with sporadic, short, and curly nanowalls, indicating that elevated heater temperatures, low microwave power, and high pressure were not indicated for the growth process. Conversely, sample cPAN4 exhibited well-developed vertical nanowalls in its upper region, while its lower portion displayed structures reminiscent of a precursor-poor growth process, suggesting that plasma exposure variability impacts nanostructure uniformity. In contrast, sample cPAN5 exhibited homogeneous nanowall-like features across all surfaces, indicating a less significant influence of plasma distribution, thereby promoting a consistent nanostructure morphology. Samples cPAN6 and cPAN7 exhibited short, bulky nanowalls and sphere-petal agglomerates, with sample cPAN7 having well-defined petal-like structures on flat surfaces and uniform petal-like structures on rough surfaces, highlighting the influence of surface topology on nanostructure formation. The analysis of the conditions that influenced these variations indicated that high heater temperatures and low plasma power generally inhibited nanowall formation, leading to sporadic, short, and bulky nanostructures. In contrast, lower temperatures and higher pressures within the chamber resulted in a homogeneous distribution of nanostructures over the surface of the scaffold. The induction of nanopetal-like morphology was observed to be most effective under low-pressure conditions (20 Torr) and at relatively low diborane concentrations.

The TEM images in Fig. 3h, i provide critical insights into the nanostructure of the B,N-doped carbon scaffolds. Figure 3h reveals the presence of bulky (red arrow) and thin (dark arrow) nanowall-like structures, which are a direct consequence of boron incorporation during the MPECVD process, which promotes the growth of ordered graphitic domains. The layered structure enhances the electrochemical surface area and facilitates rapid electron transfer, contributing to improved electrocatalytic performance. Selected area electron diffraction (SAED) measurements and high-resolution (HR) TEM analysis were performed to investigate the crystallinity of the nanostructures and their crystallographic lattice. In particular, the inset of Fig. 3h shows the SAED pattern of the sample area visible in the same figure. The diffraction spots, distributed on discontinuous rings, correspond to interplanar distances (0.337, 0.203, 0.167, and 0.118 nm) that can be associated with graphite (International Center for Diffraction Data, card n° 41–1487). Conversely, Fig. 3i presents an HR-TEM image of a folded thin nanowall-like structure, in which lattice planes are discernible (dark arrows) and their interplanar distance is approximately 0.34 nm, which is in precise agreement with the (002) interplanar distance of graphite (*d*(002) = 0.337 nm). These results indicate that the carbonaceous nanostructures are crystalline and possess a graphite lattice.

The effect of synthesis parameters on the Raman properties was systematically investigated (Fig. 3j). The D-band arises from the breathing modes of *sp*^2^ atoms in rings and is associated with the presence of defects and disorder, while the *G*-band corresponds to the in-plane stretching of *sp*^2^ carbon bonds in a graphitic material. The *D*' band (1620 cm^−1^) arises from an intravalley double resonance Raman process and is also defect activated, like the *D*-band. It is associated with defects but involves different scattering processes. While the *D*-band is generally more sensitive to a broader range of defects, including edges and *sp*^3^ hybridization, the *D*' band is particularly sensitive to point defects and disturbances in the translational symmetry [41]. A minor impact, but still evident, was provided by the change of pressure: increasing pressure during MPECVD graphene synthesis introduces stress and disorder into the graphene lattice without significantly affecting the defect concentration, due to the *G* and *D* peak blueshift which indicates compressive stress within the graphene structure and broadening of the *G* peak (Fig. 3l). However, the constant *D*'/*G* ratio and the slightly decreased *D*/*G* ratio may indicate that the decrease mainly affects defects not associated with edges or grain boundaries. Major effects were brought by the process temperature: increased synthesis temperature leads to higher strain in the graphene lattice, causing both the *G* and *D* peaks to redshift. The decrease in *D*/*G* intensity ratio and the slight decrease in *G* peak FWHM suggest that this strain increase is not accompanied by a significant increase in defect density. Moreover, increasing the CH_4_ content during MPECVD synthesis of graphene leads to a more ordered structure with fewer defects, as evidenced by the decrease in *I*_D_/*I*_G_ and *I*_D'_/*I*_G_ ratios. This is likely due to CH_4_ acting as both a carbon source and an etching agent, with an optimal concentration balancing defect formation and removal. Finally, increasing microwave power in your MPECVD process leads to more defects in the graphene, but also promotes a more ordered crystal structure. Both the *D* and *D*' peaks originate from defects, so their increase relative to the *G* peak means more disorder, but the decreasing FWHM of *G* means a more uniform and ordered crystalline structure with less variation in bond lengths and angles. This may at first sight appear to be a contraction due to the increasing disorder, but it is explained by the typical morphology of edge-rich carbon nanowalls on the scaffold surface.

A simultaneous increase in *A*(*D*) and *A*(*D*') indicates a higher density of defects within the graphite or graphene material, implying lower crystalline quality and increased disorder within the material [42]. Furthermore, the A(*D)*/A(*D*') = 10 ratio suggests that the CVD-grown sample primarily contains defects associated with *sp*^3^ hybridization, with a lesser extent of vacancy-like defects [43]. Conversely, a greater presence of edges is observed, and it is more reasonable to expect a ratio of ~ 3 relative to boundary defects. The carbonization of PAN results in substantial alterations to its chemical structure, as evidenced by the C 1*s* and O 1*s* XPS spectra (Fig. 3k, Table S5). This process leads to an increase in the proportion of *sp*^2^ hybridized carbon, as indicated by an increase in the *sp*^2^ peak intensity in the C1s spectrum. Concurrently, peaks associated with C–N and C=N bonds decrease, reflecting the loss of nitrogen. In the O 1*s* spectrum, a decrease in peak intensity is observed, corresponding to the overall reduction of oxygen content in the material. Since boron is introduced during carbonization, B–C bonds are evident in the B1s spectrum.

### Electrochemical Characterization

**Fig. 4:**
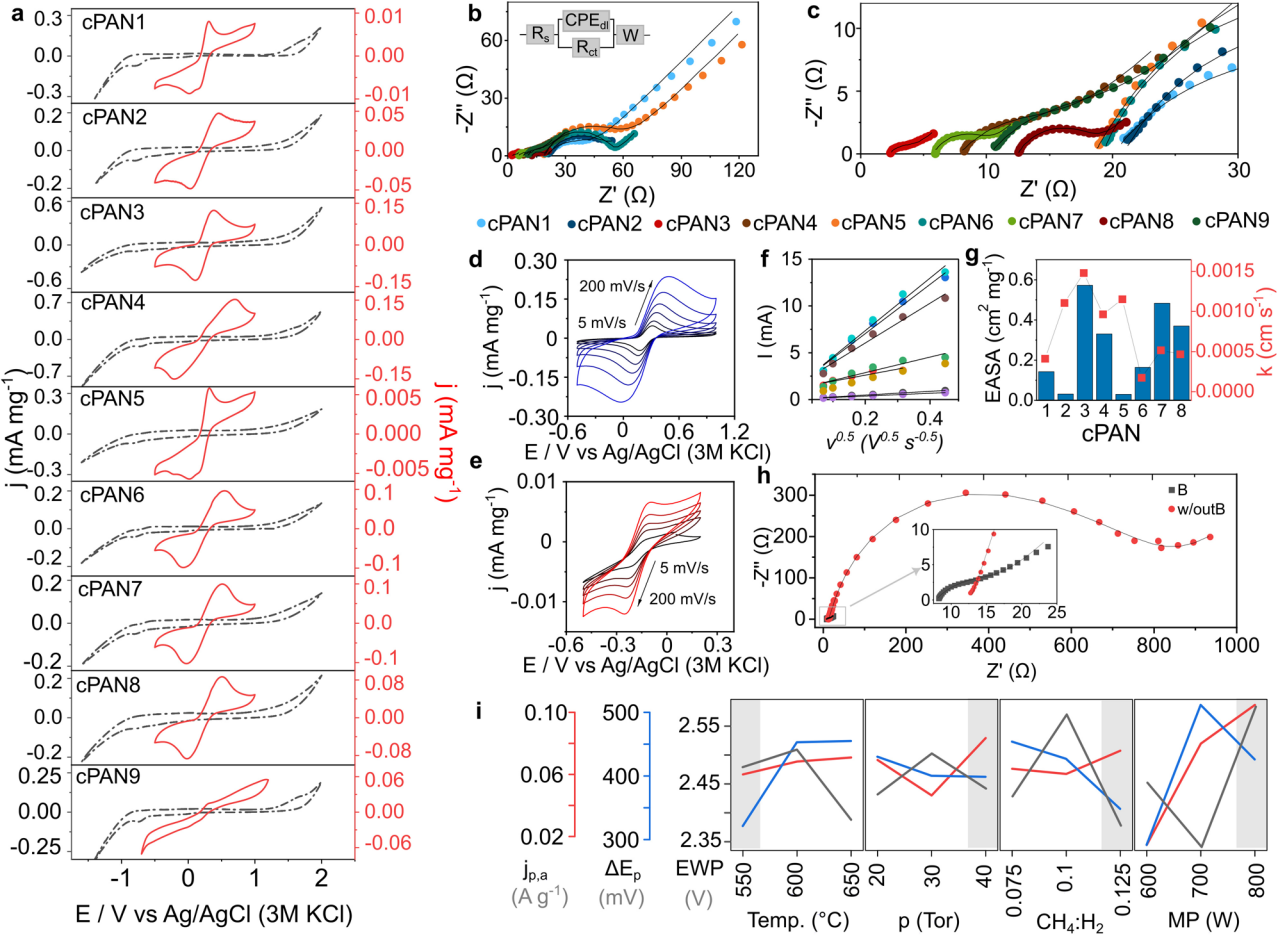
Electrode electrochemical characterization. **a** CV curves in the presence of 5 mM Fe(CN)_6_^3−/4−^ in 0.05 M PS, scan rate 50 mVs^−1^, **b**, **c** Nyquist plots, dotted lines are experimental values and solid lines are fitted, insert: the modified Randles equivalent circuit. CV curves in different scan rate: 5, 10, 25, 50, 100 and 200 mVs^−1^ in: **d** 5 mM Fe(CN)_6_^3−/4−^ in 0.05 M PS for cPAN3, **e** 5 mM Ru(NH_3_)_6_^2+/3+^ in 0.05 M PS for cPAN1. **f** Randles–Sevcik plots with anodic and cathodic slopes obtained by the linear fitting in Fe(CN)_6_^3−/4−^ redox couple with **g** EASA and heterogeneous electron transfer rate constant (k_0_) for all cPAN samples. **h** Comparison of EIS between the B-doped and undoped cPAN at the same MPECVD conditions and **i** Taguchi analysis based on Fe(CN)_6_^3−/4−^ as a redox pair

CV measurements were performed in 0.1 M PS solution (pH 7.8) at a scan rate of 50 mV s^−1^ (Fig. 4a gray line). The potential windows were determined to be approximately from 2.16 V (cPAN7) to 2.70 V (cPAN4). While the current rise beyond ~ 1.4 V vs Ag/AgCl indicates OER, MPECVD-induced surface modifications appear to shift the onsets of OER and HER, slightly broadening the electrochemical window. The potential windows are relatively wide, and a higher O_2_ overvoltage affects the efficient oxidation properties of the anode. This is a desirable characteristic, as it ensures that the supplied electrical energy is not wasted in O_2_ generation in EO processes [44]. Next, the electrochemical property of the electrodes was investigated using the ferrocyanide/ferricyanide redox couple, characterized by a one-electron reversible redox system. Resulting CV characteristics are given in Fig. 4a red line. Electrodes cPAN1, cPAN3, and cPAN5 show well-defined redox peaks and have relatively low peak-to-peak separation values (268, 265, and 264 mV, respectively), indicating faster electron transfer kinetics compared to the other electrodes. The peaks observed for cPAN9 were significantly less prominent, indicating non-uniform surface and complex reactions with varying kinetics occurring on the surface. Moreover, electrode cPAN3 has the highest anodic peak current magnitude value at 0.124 mA mg^−1^ and the anodic-to-cathodic current ratio value at 0.98 indicating near-perfect reversibility.

The higher peak current magnitude values may be related to a higher surface area and lower cPAN3 electrode material resistance than the other electrodes, due to well-developed and uniformly distributed carbon nanowalls throughout the surface. The cPAN3 electrode demonstrated enhanced electrochemical performance, which can be related to the easier adsorption of electroactive ions by the *sp*^2^ carbon. Moreover, the CV measurements were conducted at various scan rates from 5 to 200 mV s^−1^ (for electrode cPAN3, Fig. 4d, e). Figure S5a shows that the anodic and cathodic currents of the faradaic process increase linearly with the square root of the scan rate, proving that redox processes are limited by diffusion. The symmetry between the anodic and cathodic reactions suggests that the electron transfer process is almost reversible. The slope values of the logarithm of anodic and cathodic peak current densities versus the logarithm of the scan rate are equal to 0.390 (*R*^2^ = 0.996) and 0.407 (*R*^2^ = 0.997), respectively, which is near the theoretical value of 0.5 for the diffusion-controlled process (Fig. S5b). In case of other samples, the correlation between the logarithms of anodic peak current magnitude and scan rate for Fe(CN)_6_^3−/4−^ are presented in Fig. S6, obtained from the CV curves in Fig. S7, and the slope values (b- value) are reported in Table S6. The calculated anodic slope values range from 0.298 (cPAN2) to 0.424 (cPAN1) with high linearity dependency, suggesting diffusion limited processes for each of modifications. The Ru(NH_3_)_6_^2^⁺/^3^⁺ redox couple also shows linear dependence of anodic/cathodic current on the square root of the scan rate (Fig. S5c), also indicating diffusion control. However, in the Ru(NH_3_)_6_^2+/3+^ we observed discrepancy between the highest anodic and cathodic peak current value (Fig. S5d) indicating less reversible electron charge transfer than Fe(CN)_6_^3−/4−^.

The impedance measurements were performed in 5 mM Fe(CN)_6_^3−/4−^ in 0.05 M phosphate buffer solution (PBS) (Fig. 4b, c). Considering the complex and heterogeneous 3D structure of the electrodes, the EIS study was modeled using modified Randles electrical equivalent circuit. This equivalent circuit includes the ohmic resistance of the electrolyte (*R*_s_), the charge transfer resistance (*R*_ct_), the electrochemical double-layer capacitance (*C*_dl_), and the Warburg impedance (*Z*_w_). The equivalent electrical circuit is shown in the inset of Fig. 4b, and the *R*_ct_ fitted values, together with the electroactive surface area (EASA), are reported in Table S7. The Nyquist spectra for the electrodes present the shape of a flattened semicircle in the high-frequency part and a straight Warburg line at low frequency. The semicircle's diameter is related to the charge transfer resistance at the electrode/electrolyte interface and the pores resistance. The straight lines are characteristic of the Warburg impedance, resulting from the diffusion of charged species from the bulk of the electrolyte solution to the interface and via the interface layer. The charge transfer resistance values range from 1.7 Ω for cPAN3 to 38.8 Ω for cPAN5, indicating that cPAN3 exhibits the most efficient electron transfer and thus the superior electrochemical performance (Table S7). Among the samples, cPAN3 exhibits the highest EASA of 0.57 cm^2^ mg^−1^ and the highest heterogeneous electron transfer rate constant (*k*_0_) of 0.00159 (Fig. 4g). The significant enhancement in charge transfer, attributed to boron doping, is further highlighted in Fig. 4h. All samples analyzed in this study were synthesized with boron doping via B_2_H_6_, except for the control sample (red markers), which was intentionally grown without B_2_H_6_ to isolate the effect of boron incorporation.

Additionally, double-layer capacitance was estimated from cyclic voltammetry in the non-Faradaic potential range in 1 M NaCl aqueous electrolyte at various scan rates (Fig. S8). In contrast with other samples, cPAN3 shows a significantly higher *C*_dl_ of 57.0 µF mg^−1^, with high linearity dependency (Fig. S9 and Table S8), demonstrating its superiority in capacitive performance. This finding is consistent with elevated EASA for cPAN3, demonstrating a highly developed and accessible surface.

The Taguchi experiment design was employed to optimize key parameters, including microwave power, temperature, gas flow, and pressure, each set at three levels. An orthogonal array was used to test different combinations of these factors, with experiments conducted randomly according to the L9 array. Performance was evaluated using relevant metrics, as shown in Fig. 4j, and a main effect plot was generated to assess the impact of each factor. The analysis revealed that the lowest temperature, low CH_4_/H_2_ gas precursor composition, highest pressure, and microwave power were most effective in achieving the widest electrochemical potential windows. Similar trends were observed for temperature and pressure regarding the lowest peak-to-peak separation of the ferro-ferricyanide redox couple, while opposite trends were noted for CH_4_/H_2_ composition and microwave power. The optimal parameters identified through this analysis resulted in the formation of a homogeneous carbon nanowalls covering the scaffold surface. Based on these results, the selected conditions were: temperature: 550 °C, pressure: 40 Torr, CH_4_: 20 sccm, and microwave power: 700 W.

### Electrochemical Oxidation of β-Blockers

**Fig. 5:**
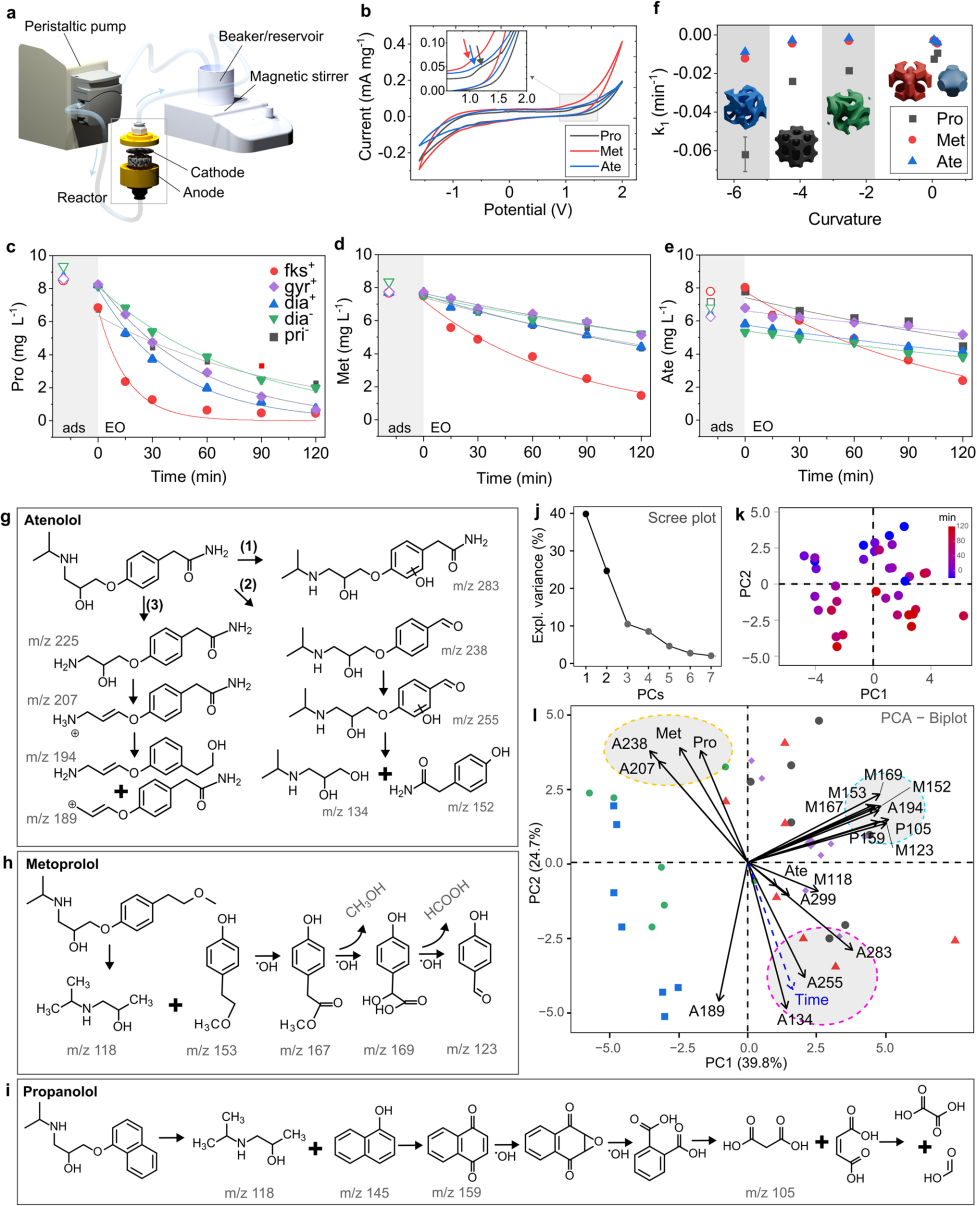
EO test results. **a** Schematic of the experimental setup. **b** CV of the electrode in the presence of Pro, Met, and Ate and **c**–**e** their concentration during the EO tests. **f** Correlation between first-order kinetic oxidation of Pro, Met, and Ate and TPMS curvature. **g**–**i** Proposed degradation pathway and PCA results: **j** scree, **k** score plots (with time dependence highlighted) and **l** biplot

The integration of structurally complex materials through techniques such as electrospinning and 3D printing has proved to significantly enhance performance in energy-related applications. For instance, the combination of NiCoP and MXene in 3D-printed architectures has resulted in supercapacitor electrodes with superior areal and volumetric capacitance [3]. Similarly, printable energy storage strategies using tailored ink and filament formulations and pore-engineered scaffolds have facilitated enhanced ion transport and optimized electrode performance [4]. Electrode performance was evaluated as the anode in the electrochemical oxidation of Pro, Met, and Ate. β-adrenergic receptor antagonists (β-blockers) such as atenolol, metoprolol, and propranolol are commonly used pharmaceuticals that have been detected in wastewater and surface water due to their widespread use and persistence [45] and may harm different organisms [46]. Previous studies have reported their degradation through advanced oxidation processes, including electrochemical oxidation, where hydroxyl radicals (•OH) play a key role in breaking down these compounds into smaller intermediates [46, 47]. Figure 5a shows electrochemical oxidation (EO) setup, including a peristaltic pump for fluid circulation, a beaker (reservoir) with a magnetic stirrer, and an electrochemical reactor containing the cathode and anode. The electrochemical oxidation experiments were conducted in a flow-through reactor, with the working electrode serving as the anode. The anode is a porous cylindrical electrode with a 25 cm diameter and 8 mm thickness. The cathode consists of a 316L stainless steel mesh, which is separated from the anode by a 1-mm-thick flexible silicone separator to prevent direct contact while maintaining efficient ion transport. The flow direction in the reactor is bottom to top, ensuring uniform flow distribution and facilitates the dispersal of bubbles. The power supply used for all electrochemical tests was the potentiostat, as described in Materials section.

Figure 5b shows the electrochemical behavior for the three compounds. The inset zooms in on a region around 0.5–2 V, showing distinct behaviors of Pro (black line), Met (red line), and Ate (blue line). The electrochemical response suggests different degradation potentials for each compound. While a peak appears for Met and Pro, Ate is not directly oxidized. Figure 5c–e shows the degradation of Pro, Met, and Ate during EO using different electrode geometric structures. The compounds are monitored over 120 min, and their concentrations decrease effectively over time. The degradation rates depend on the geometric structure of the material used as the electrode. The electrode geometry fks^+^ performs the best in degrading β-blockers, as it shows the fastest decrease over time, while other geometries such as gyr^+^ or dia^+^, dia^−^, and pri^−^ have a slower degradation kinetic. Figure 5f compares the first-order degradation rate constants for Pro, Met, and Ate across different electrode geometries, as a function of curvature, as determined by CFD simulations. The fastest degradation reactions are observed for Pro, which was also emphasized by Kovács et al. (2022) [47]. The performance of the studied electrodes, especially fks^−^, compared with other carbon-based electrodes, demonstrates comparable efficiency for β-blockers removal (75%–99.9%) [48–54]. Notably, several studies report enhanced performance upon the application of sulfate-based electrolytes. A detailed performance comparison can be found in the Supplementary Materials (Table S9). The identification of transformation products is crucial for assessing the environmental impact of electrochemical oxidation. Some intermediate compounds, such as quinones and aldehydes, may exhibit higher toxicity than the parent compounds, requiring additional treatment to ensure complete mineralization into non-toxic end products like CO_2_ and H_2_O. Monitored degradation products by HPLC–MS/MS using SIM mode are reported in Table S10. Proposed degradation pathways for Ate, Met, and Pro are presented in Fig. 5g–i, respectively. Ate, Met, and Pro are structurally similar compounds, both featuring an oxypropanolamine side chain with an amino group and a hydroxyl (OH) group. Hence, the same transformation products may occur (e.g., m/z 118 in Met and Pro). Degradation products of Ate (Fig. 5g) may undergo multiple transformation mechanisms simultaneously (pathways 1–3). Formation of A283 occurs via hydroxylation (pathway 1), but mass spectrometry data do not allow identification of the site of preferential attacks by the radicals or the exact position of hydroxylation. However, either the ortho- or meta-position on the aromatic ring were likely reactive sites for hydroxyl group addition [55, 56], e.g., 4-[2-hydroxy-3-[(1-methylethyl)amino]propoxy]benzamide. Considering pathway (2), amide bond cleavage, hydroxylation, and ether bond scission are observed. Loss of the amide group followed by the addition of oxygen to the alkyl group and hydrogen atom transfer by •OH resulted in the formation of an intermediate A238, identified as (4-[2-hydroxyl-3-(isopropylamino)propoxy]benzaldehyde) [56]. Intermediate m/z 225 formed as a result of further hydroxylation, and A134 and A152 as ether bond scission. In pathway (3), isopropyl cleavage leads to the formation of A225. Then, A225 can be oxidized to form A207, which can undergo subsequent oxidation to give A194 and A189.

Met (Fig. 5h) undergoes degradation mainly through a radical attack on the aromatic ether, breaking the C-O bond and releasing 3-(propan-2-ylamino)propan-1,2-dion (M118) along with 4-(2-methoxyethyl)phenol (M153). Further radical cleavage of 4-(2-methoxyethyl)phenol leads to the formation of methyl 4-hydroxyphenylacetate (M167). This compound is subsequently hydroxylated, producing 2-hydroxy-2-(4-hydroxyphenyl)acetic acid (V), accompanied by the release of methanol (MeOH). The oxidation of (M167) progresses to form 4-hydroxybenzaldehyde (M123), releasing formic acid. Further cleavage of the benzene ring in the product may result in the formation of smaller compounds, such as short-chain carboxylic acids [57]. The initial hydroxylation of Pro (Fig. 5i) may lead to the formation of 1-naphthol (P145); however, it was not found under the presented study. Nevertheless, its oxidation products 1,4-naphthoquinone (P159), as well as further transformation product: glycolic acid (P105), were observed. According to Isarain-Chávez et al. (2011), primary 1-naphthol can be degraded into phthalic acid, suggesting that naphthalene compounds are first oxidized to benzene derivatives. Subsequently, final carboxylic acids, such as maleic, oxalic, and formic acids, are produced during the degradation of both types of aromatic compounds [58]. The PCA analysis, combined with the HPLC–MS/MS results, provides critical insights into the degradation pathways of atenolol (Ate), metoprolol (Met), and propranolol (Pro) (Fig. 5j–l). While Met and Pro degradation products (A238, A207) exhibit a clear inverse correlation with time, the Ate by-products (A255, A134, A283, A189) increase over time, indicating incomplete mineralization regardless of the degradation route (1), (2), or (3). This difference suggests that Ate may follow an alternative degradation mechanism distinct from Met and Pro, despite their structural similarity. The clustering of all Met transformation products (except for M188) suggests that these compounds follow a common degradation pathway mediated by •OH radicals, while M188 may arise through a separate mechanism. Additionally, the presence of P159 in the same cluster further supports the hypothesis that these products are primarily linked to hydroxyl radical-driven reactions. Another significant insight from the PCA is the identification of a group of byproducts (M167, M153, M169, M152, A194, P105, M123, P159) that remain uncorrelated with time. This implies the existence of secondary degradation processes independent of the primary radical-driven pathways. Such findings highlight the complexity of the degradation mechanisms and emphasize the role of •OH radicals in driving these transformations. The PCA also suggests that the Ate degradation pathway may involve additional steps beyond the initial radical attack, differentiating it from Met and Pro.

## Conclusions

This study presents a novel and scalable strategy to fabricate 3D-printed carbon electrodes with hierarchical porosity by integrating topology optimization, phase inversion-assisted micromolding, and MPECVD. Through systematic material and process optimization, we demonstrate that porosity, nanostructure growth, and electrochemical properties can be finely controlled to improve the performance of electrochemical oxidation (EO) electrodes for wastewater treatment. We found that microscale porosity is tuned by mold chemistry, rather than by different dissolution kinetics. Specifically, the use of BVOH instead of PVA increased surface roughness and localized porosity near the electrode surface, while the addition of cosolvents (acetone) promoted diffuse micron-scale porosity. This tailored porosity alleviates shrinkage-induced stress during thermal treatments, preserves the fidelity of the electrode geometry, and reduces deformation from the original model. The nanostructure morphology of the electrode surface was optimized by tuning the MPECVD synthesis parameters, resulting in the growth of vertically oriented graphitic sheets with superior electrochemical properties. The optimal MPECVD conditions resulted in an 8.5-fold increase in the electrochemically active surface area (EASA) and a 6.2-fold increase in the charge transfer rate constant (*k*₀) compared to non-optimized structures. In addition, our analysis revealed that the degradation pathways of metoprolol and propranolol follow similar reaction mechanisms, whereas atenolol undergoes a distinct transformation pathway, suggesting different oxidation intermediates and selectivity. The highly porous, conductive, and catalytically active electrodes developed in this study show significant potential for scalable, metal–catalyst-free electrochemical water treatment. This approach enhances pollutant degradation efficiency while reducing reliance on critical raw materials, offering a promising and sustainable alternative for advanced wastewater treatment technologies.

## Supplementary Information

Supplementary file 1

Supplementary file 2
