# Supplementary material for: 3D-Printed Boron-Nitrogen Doped Carbon Electrodes for Sustainable Wastewater Treatment via MPECVD
Source: Nanomicro Lett. 2025 Jun 24;17:311. doi: 10.1007/s40820-025-01827-9 (PMC12185841; doi:10.1007/s40820-025-01827-9)
Supplement: Supplementary file 1 [file 40820_2025_1827_MOESM1_ESM.docx]

Supporting Information for

**Topologically Optimized, Mesostructured Carbon Electrodes for Enhanced Mass Transport and Reaction Kinetics**

Iwona Kaczmarzyk^1^, Malgorzata Szopińska^2^, Patryk Sokołowski^1^, Simona Sabbatini^3^, Gabriel Strugala^4^, Jacek Ryl^5^, Gianni Barucca^3^, Per Falås^6^, Robert Bogdanowicz^1^, Mattia Pierpaoli^1,*^

^1^ Faculty of Electronics, Telecommunications and Informatics, Gdansk University of Technology, 11/12 Gabriela Narutowicza Street, Gdansk (80-233), Poland

^2^ Faculty of Civil and Environmental Engineering, Gdansk University of Technology, 11/12 Gabriela Narutowicza Street, Gdansk (80-233), Poland

^3^ Department of Materials, Environmental Sciences and Urban Planning (SIMAU), Università Politecnica delle Marche, INSTM Research Unit, Via Brecce Bianche 12, Ancona (60131), Italy

^4^ Department of Materials Science and Technology, Institute of Manufacturing and Materials Technology, Faculty of Mechanical Engineering and Ship Technology, Gdańsk University of Technology, 11/12 Gabriela Narutowicza Street, Gdansk (80-233), Poland

^5^ Division of Electrochemistry and Surface Physical Chemistry, Faculty of Applied Physics and Mathematics, Gdańsk University of Technology, Narutowicza 11/12, Gdansk (80-233), Poland

^6^ Division of Chemical Engineering, Department of Process and Life Science Engineering, Lund University, PO Box 124, SE-221 00 Lund, Sweden

*Corresponding author. E-mail: [mattia.pierpaoli@pg.edu.pl](mailto:mattia.pierpaoli@pg.edu.pl) (Mattia Pierpaoli)

# **S1 Materials and methods**

Reynolds number is a dimensionless quantity that compares inertial forces to viscous forces within a fluid. It's calculated using the fluid density, average velocity in the flow direction, hydraulic diameter, and fluid viscosity.

Re = (ρ * v * d_h_) / μ

where ρ is the density of the fluid (kg/m³), v the average fluid velocity (m/s), μ, the dynamic viscosity of the fluid (Pa·s) and d_h_ the hydraulic diameter (m).

**S1.1 Details on the sample preparation**

50 ml of sample diluted (1:50) of sample was use for analysis. Solid phase extraction (SPE) has been applied as a sample preparation step, and no filtration step before SPE was performed. Hydrophilic-Lipophilic Balanced – HLB, 500 mg (Oasis, Water Company) SPE cartridges was used. As a first step cartridges were conditioned using 6 ml of MeOH and 6 ml of MS-Grade water at flow rate at 1 mLmin^-1^ (gravity elution). Then 50 ml of sample spiked with surrogate standard (100 mL of Atenolol D7 and Metroprolol D7 at concentration 1 ng/mL). Moreover 100 mL of 10% FoA and 100 mL of 10% EDTA was added to the sample before being load on SPE column. Afterwards cardrige was dried under vaccum for 30 min to remove excess water. Elution of the target analytes was performed with 2 * 3ml of MeOH at 1 mLmin^-1^. Extracts were evaporated to dryness under the gentle stream of nitrogen and the sample was reconstituted with 1000 mL of MeOH and stirred 60 s for the proper mixing.

As a final step 1 ml sample was filtrated using polytetrafluoroethylene (Chromafil PTFE- 20/15 MS, Macherey-Nagel, Germany) 0.2 µm pore size syringe filters. 1mL of reconstrued sample was directed to UHPLC-ESI-MS/MS analysis.

**S1.2 UHPLC-ESI-MS/MS method description**

An ultra-high performance liquid chromatography tandem mass spectrometry with electrospray ionization (UHPLC-ESI-MS/MS) Nexera XR coupled with LC/MS-8050, Shimadzu Company) was used to determined selected b-blokers: metoprolol, propranolol, atenolol and their degradation products.

Shim-pack SP-C18, 2.1 × 150 mm, and 2.7 µm was used as an analytical column. A constant flow was applied at 0.4 mlmin^-1^. A gradient mobile phase was used; starting at 90% mobile phase A (5mM ammonium formate and 0.02% formic acid) in ultrapure water), and 10% of mobile phase B (ACN and 0.02% formic acid) kept 4.5 min, followed by a linear increase over 15min to 10% A and 90% B, then kept through 2 min (till 17 min) and then returned to the initial conditions (1 min mark), and held for 2 min (re-equilibration to initial condition). The column temperature was maintained at 40 °C and the autosampler at 4 °C. The total run time was 20 min. LC eluate was sprayed into a tandem mass spectrometer by electrospray ionization in the positive ion mode. The interface setup parameters were as follows: nebulizing gas flow (N2, 3 Lmin^-1^), heating gas flow (N2, 10 Lmin^-1^), interface temperature (150 °C), desolvation temperature (261 °C), heat block temperature (400 °C).

MET, ATE and PROP were determined using MRM mode with the following transitions: Atenolol 267.1500>145.0500, 267.1500>190.0500, 267.1500>74.1000; Atenolol D7 274.1500>145.0000, 274.1500>190.0000; Metoprolol D7 275.1500>123.1500, 275.1500>79.1500, 275.1500>105.1500; Metoprolol 268.2000>116.1000, 268.2000>74.1000, 268.2000>133.0500; Propranolol 260.1500>116.0500, 260.1500>183.0500, 260.1500>56.1000 in the positive ionization mode (electrospray ionization – ESI). Retention time for this compound were following: MET – 7.030 min; PROP – 8.380 min; ATE – 0.883 min; ATE D7 – 0.847 min (internal standard for ATE); MET D7 – 7.060 (internal standard for MET I PROP). The calibration curves for ATE, MET and PROP were plotted using 5-level calibration (linearity in the ranges of 1–10 ngmL^-1^: ATE r^2^=0.993; MET r^2^= 0.993; PROP r2= 0.995). In this method relative recoveries were 77.13%, 71.4% and 76.8 % for MET, ATE and PROP, respectively. Precision measured as a variation coefficient was 3.38% 12.18% and 9.6% for MET, ATE and PROP, respectively.

**Supplementary Tables and Figures**

**Table S1** TPMS equations

| **Primitive** | **Equation** | |
| --- | --- | --- |
| Diamond | $f=\cos\left( X \right)\cos\left( Y \right)\cos\left( Z \right)-\sin\left( X \right)\sin\left( Y \right)sin(Z)$ |  |
| Fischer-Koch S | $f=(cos \left( X \right)\cos\left( Y \right)+\cos\left( Y \right)cos(Z))-(\cos\left( 2X \right)+\cos\left( 2Y \right)+cos(2Z))$ |  |
| Gyroid | $f=\sin\left( X \right)\cos\left( Y \right)+\sin\left( Z \right)\cos\left( Z \right)+\sin\left( Y \right)cos(Z)$ |  |
| Schoen's I-WP | $f=2\left( \cos\left( X \right)\cos\left( Y \right) \right)+\cos\left( Y \right)\cos\left( Z \right)+\cos\left( Z \right)cos(X))-(\cos\left( 2X \right)+\cos\left( 2Y \right)+cos(2Z))$ |  |
| Neovius | $f=3\left( \cos\left( X \right)+\cos\left( Y \right)+\cos\left( Z \right) \right)+4(\cos\left( X \right)\cos\left( Y \right)\cos\left( Z \right))$ |  |
| Schwarz P-Surface | $f=cos(X)+cos(Y)+cos(Z)$ |  |

**Table S2** Filament description and printing parameters

| **Filament name** | **Producer** | **Extruder temp. (⁰C)** | | **Print bed temp. (⁰C)** | **Printing speed (mm/s)** |
| --- | --- | --- | --- | --- | --- |
| PVA | F3D FILAMENT  (Finnotech Sp.zoo.) | 208 | 52 | | 25 |
| C1 | AquaSolve PVA (FormFutura^®^) | 200 | 60 | | 25 |
| C2 | Atlas Support (FormFutura^®^) | 200 | 60 | | 25 |
| C3 | Helios Support (FormFutura^®^) | 240 | 65 | | 25 |
| BVOH | Fiberlogy (Fiberlab S.A.) | 212 | 60 | | 25 |

**Table S3** Process parameters

| **Sample** | **Temperature (⁰C)** | **Pressure (Tor)** | **MW power (W)** | **[CH_4_]:[H_2_]** |
| --- | --- | --- | --- | --- |
| cPAN1 | 550 | 20 | 500 | 0.075 |
| cPAN2 | 550 | 30 | 600 | 0.1 |
| cPAN3 | 550 | 40 | 700 | 0.125 |
| cPAN4 | 600 | 20 | 700 | 0.1 |
| cPAN5 | 600 | 30 | 500 | 0.125 |
| cPAN6 | 600 | 40 | 600 | 0.075 |
| cPAN7 | 650 | 20 | 600 | 0.125 |
| cPAN8 | 650 | 30 | 700 | 0.075 |
| cPAN9 | 650 | 40 | 500 | 0.1 |
|  |  |  |  |  |

**Table S4** molded PAN characterization

|  | **EDX* (at%, median value n=3)** | | | | | | | **FTIR** | **Raman** |
| --- | --- | --- | --- | --- | --- | --- | --- | --- | --- |
| **Mold** | **O** | **C** | **N** | **Al** | **Ni** | **N/C** | **C/O** | **PVA/PAN**** | **I_D_/I_G_** |
| PAN-only | 52.5 | 17.3 | 29.4 | 0.56 | - | 1.70 | 0.33 | 0.11 | 3.1 |
| PVA | 61.0 | 17.1 | 21.2 | 0.57 | - | 1.24 | 0.28 | 1.0 | 2.6 |
| C1 | 58.7 | 16.6 | 24.5 | 0.43 | - | 1.47 | 0.28 | 1.1 | 3.2 |
| C2 | 60.2 | 25.1 | 13.7 | 0.61 | 0.24 | 0.55 | 0.42 | 1.2 | 2.8 |
| C3 | 56.0 | 24.4 | 15.5 | 2.8 | - | 0.63 | 0.44 | 1.3 | 2.8 |
| BVOH | 56.2 | 24.7 | 15.8 | 3.5 | - | 0.64 | 0.44 | 1.1 | 2.9 |

**EDX was performed on the stabilized PAN (sPAN samples)*

***PVA/PAN = PVA (2244cm^−1^) / PAN (1740cm^-1^) (FTIR). Both FTIR and Raman refer to the molded PAN, prior stabilization process.*


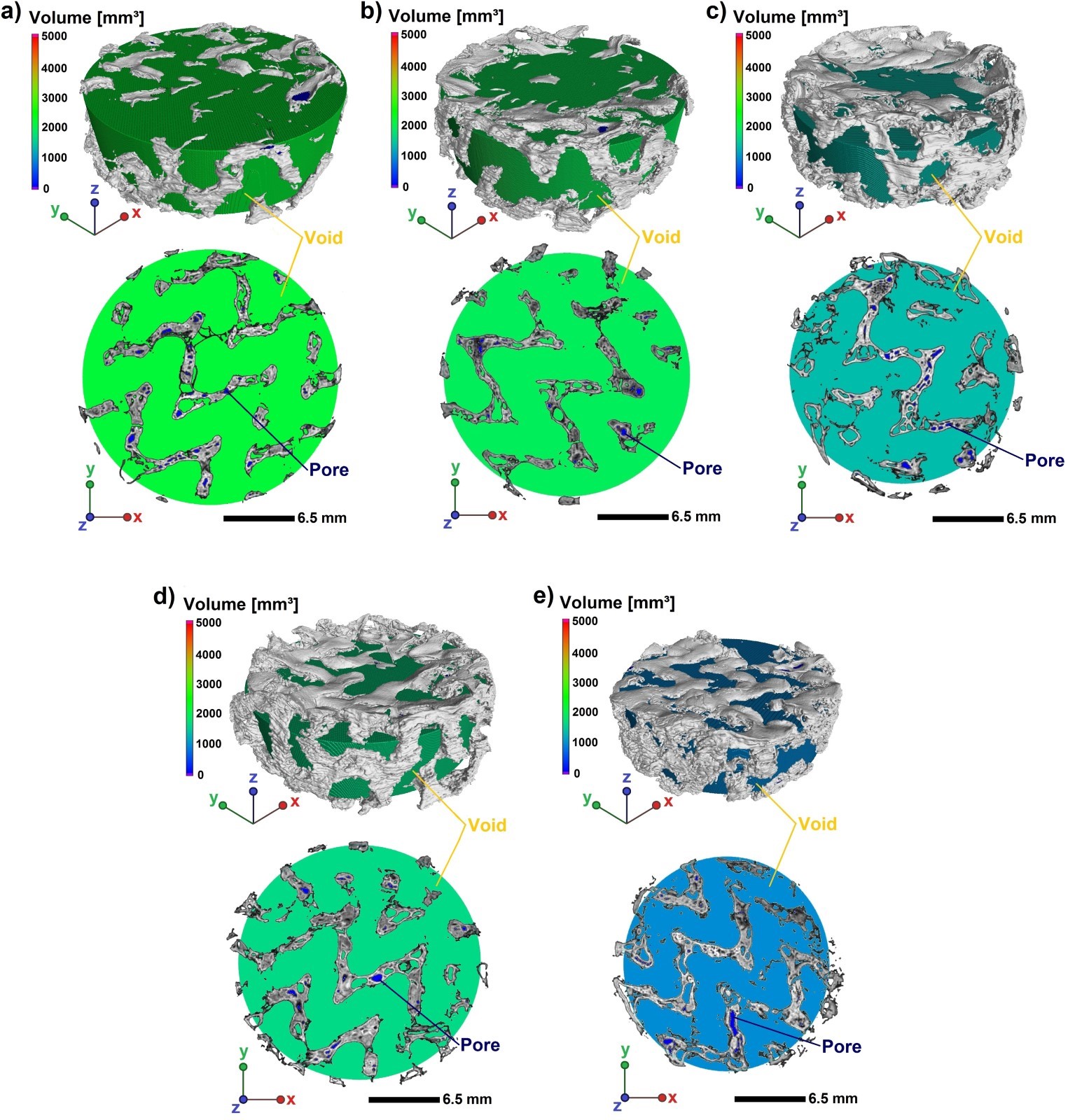


**Fig. S1** 3D visualizations and YX cross-sections of µCT reconstructed samples with voids and pores marked where: **a**) PVA, **b**) C1, **c**) C2, **d**) C3, **e**) BVOH


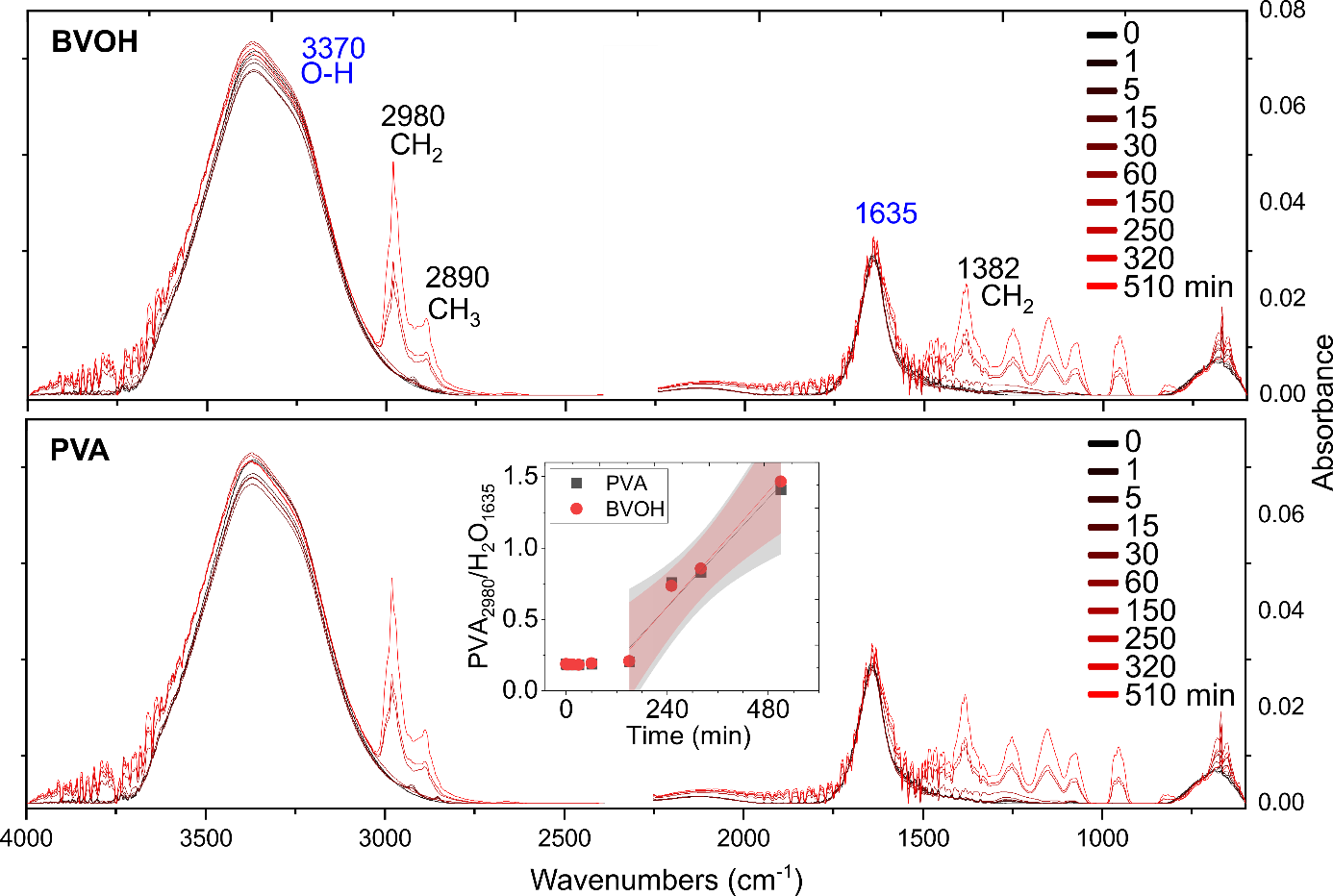


**Fig. S2** FTIR spectra of the solution during polymer dissolution


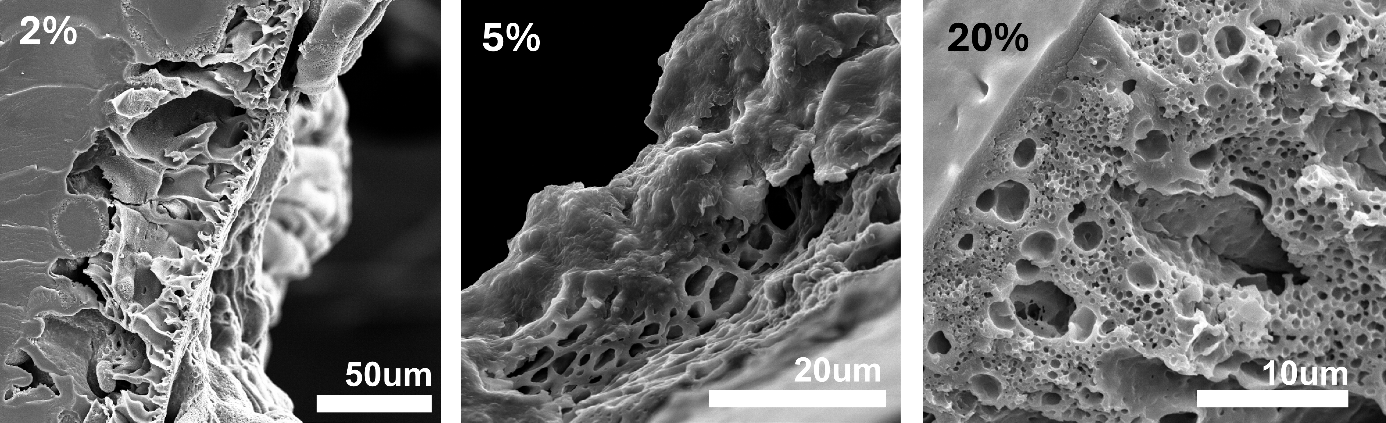


**Fig. S3** Effect of the cosolvent (acetone) to the polymer porosity


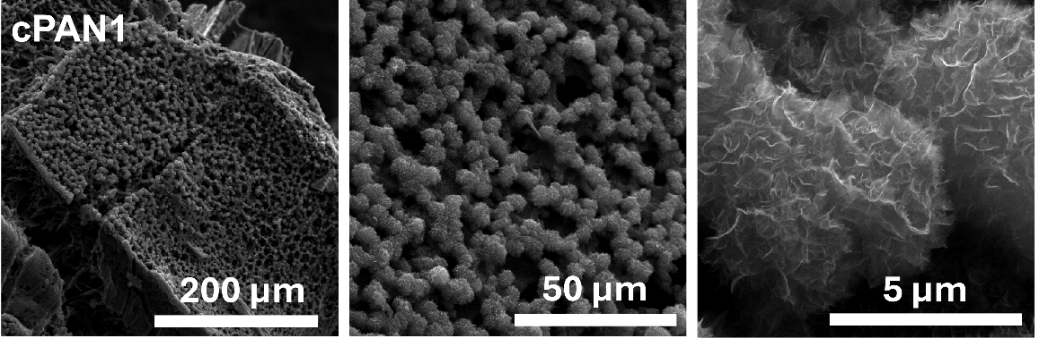


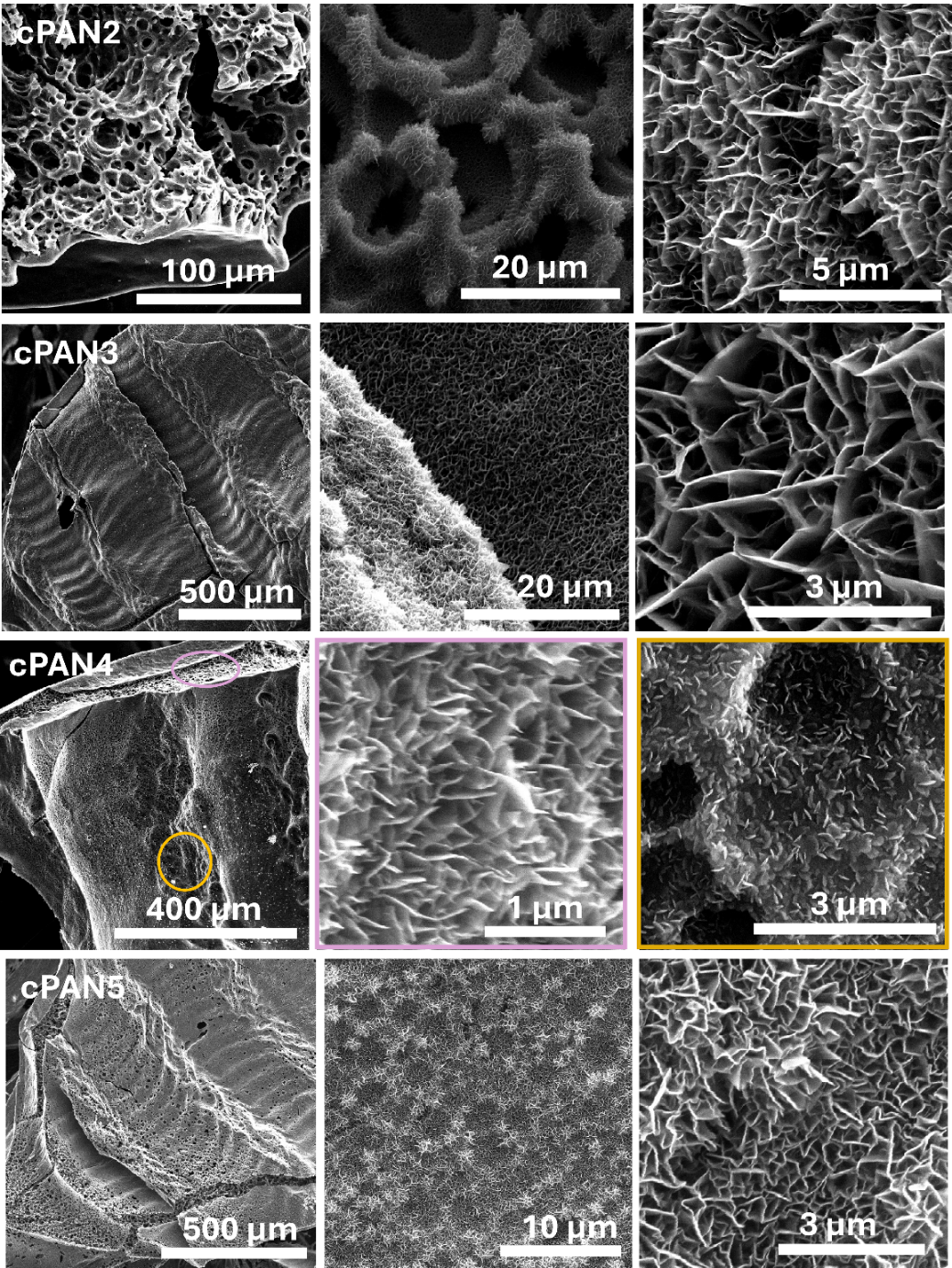


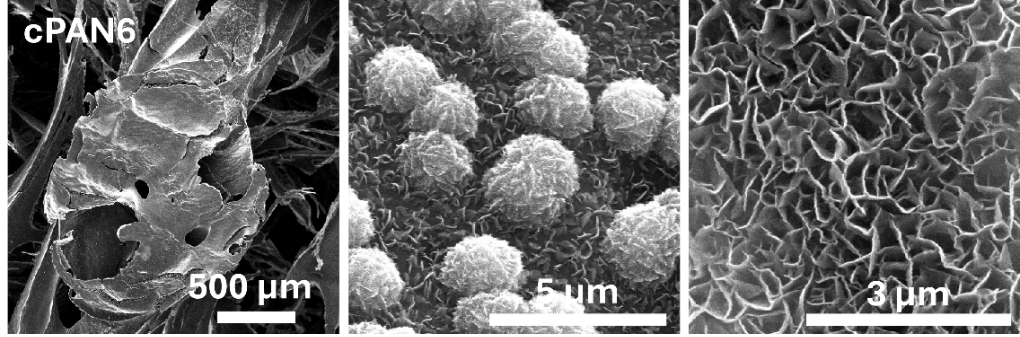


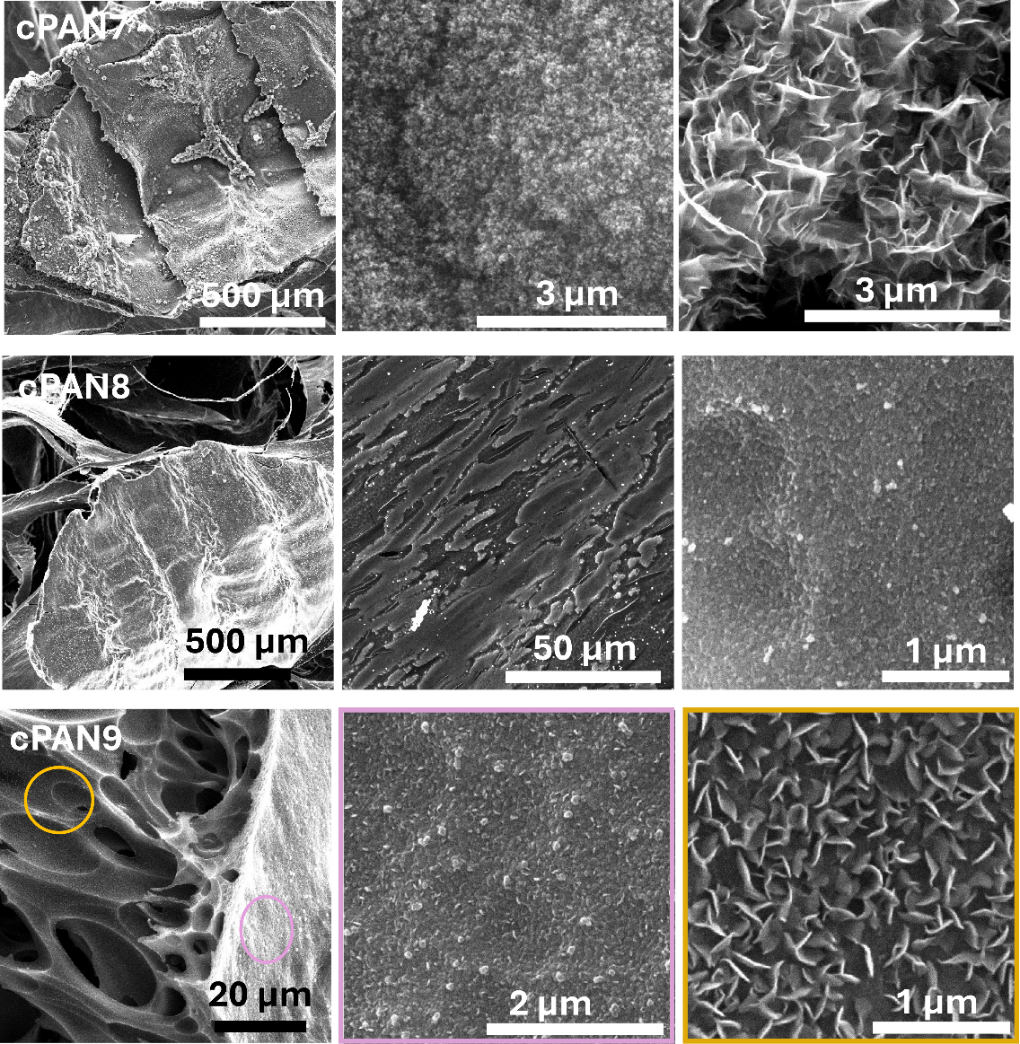


**Fig. S4** SEM image of the cPAN

**Table S5** XPS reference table with N 1s and C 1s binding energies

|  |  |  |  | **mPAN** | **sPAN** | **cPAN** |
| --- | --- | --- | --- | --- | --- | --- |
| C1s | C1 | C=C |  | 15.9% (283.6 eV) | 1.4% (283.2 eV) | 2.2% (283.2 eV) |
|  | C2 | C-C |  | 47.1% (284.9eV) | 34.0% (284.7eV) | 76.6% (284.8 eV) |
|  | C3 | C-O/C=N |  |  | 10.4% (286.0 eV) | 8.4% (285.7 eV) |
|  | C4 | –C≡N |  | 12.3% (286.6 eV) |  |  |
|  | C5 | C=O |  |  | 12.6% (286.9 eV) | 10% (287.1 eV) |
|  | C6 | COOR |  |  | 8.3% (288.4eV) |  |
|  |  |  |  |  |  |  |
| N1s | N1 | N≡C |  | 20.6% (398eV) |  |  |
|  | N2 | N=C=C |  |  | 9.4% (398.6 eV) |  |
|  | N3 | pyrrolic N |  | 2.2% (400.2eV) | 7.5% (400.0 eV) |  |
|  | N4 | graphitic N |  |  | 2.1% (401.8 eV) |  |
|  |  |  |  |  |  |  |
| O1s | O1 | O=C |  | 1.2% (529.9 eV) | 5.5% (530.8 eV) |  |
|  | O2 | O-C |  | 0.7% (531.7 eV) | 5.0% (532.0 eV) | 1% (532.4 eV) |
|  | O3 | -O=C-O |  |  | 3.7% (533.7 eV) | 1% (533.7 eV) |
|  |  |  |  |  |  |  |
| B1s | B1 | BC3 |  |  |  | 0.6% (186.9 eV) |


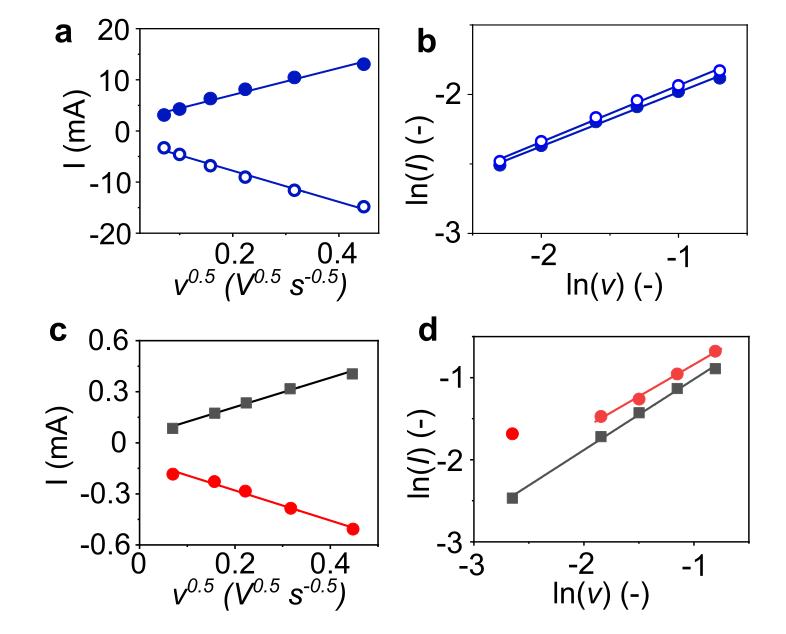


**Fig. S5** Randles-Sevcik plots with anodic and cathodic slopes obtained by the linear fitting: **a** Fe(CN)_6_^3-/4-^, **c** Ru(NH_3_)_6_^2+/3+^, the correlation between the logarithms of current density and scan rate: **b** Fe(CN)_6_^3-/4-^, **d** Ru(NH_3_)_6_^2+/3+^


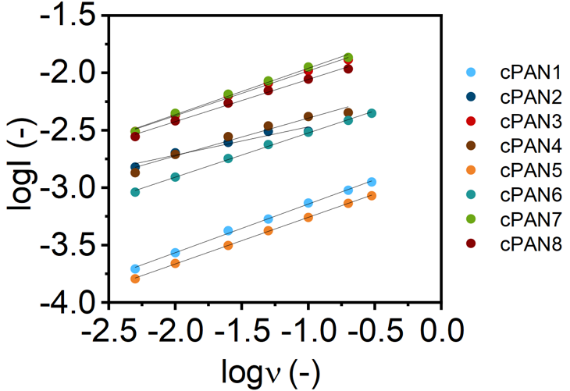


**Fig. S6** The correlation between the logarithms of anodic peak current magnitude and scan rate for Fe(CN)_6_^3-/4-^

**Table S6** Calculated b-value for Fe(CN)_6_^3-/4-^ redox probe

| **Sample** | **b - value** | **R^2^** |
| --- | --- | --- |
| cPAN1 | 0.424 | 0.9981 |
| cPAN2 | 0.298 | 0.9862 |
| cPAN3 | 0.390 | 0.9956 |
| cPAN4 | 0.330 | 0.9630 |
| cPAN5 | 0.406 | 0.9997 |
| cPAN6 | 0.385 | 0.9927 |
| cPAN7 | 0.404 | 0.9909 |

**Table S7** - Electrochemical parameters obtained by CV (scan rate 50 mV/s) and EIS, in the presence of 5 mM Fe(CN)63-/4- in 0.05 M PS

| **Electrode** | **EPW (0.1M PS)**  (V) | **D_Ep_**  (mV) | **j_p,a_**  (mA/mg) | **j_p,a_ / j_p,c_**  (-) | **R_ct_**  (Ω) | **EASA**  (cm^2^ mg^-1^) |
| --- | --- | --- | --- | --- | --- | --- |
| cPAN1 | 2.41 | 268 | 0.008 | 1.08 | 21.5 | 0.14 |
| cPAN2 | 2.51 | 431 | 0.047 | 0.98 | 29.8 | 0.03 |
| cPAN3 | 2.50 | 265 | 0.124 | 0.98 | 1.7 | 0.57 |
| cPAN4 | 2.70 | 507 | 0.103 | 0.97 | 8.1 | 0.33 |
| cPAN5 | 2.46 | 264 | 0.005 | 1.03 | 38.0 | 0.03 |
| cPAN6 | 2.34 | 588 | 0.098 | 0.98 | 35.0 | 0.16 |
| cPAN7 | 2.16 | 516 | 0.091 | 1.06 | 4.1 | 0.48 |
| cPAN8 | 2.52 | 506 | 0.087 | 1.00 | 5.9 | 0.37 |
| cPAN9 | 2.47 | - | - | - | - | - |


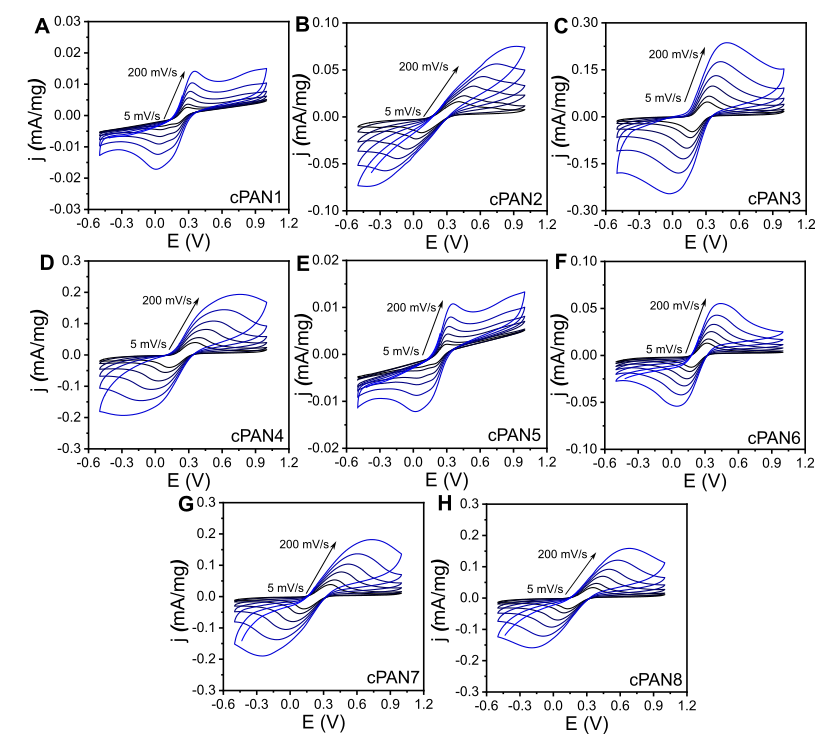


**Fig. S7** CV curves in different scan rate: 5, 10, 25, 50, 100 and 200 mVs^-1^ in 5 mM Fe(CN)_6_^3-/4-^ in 0.05 M PS


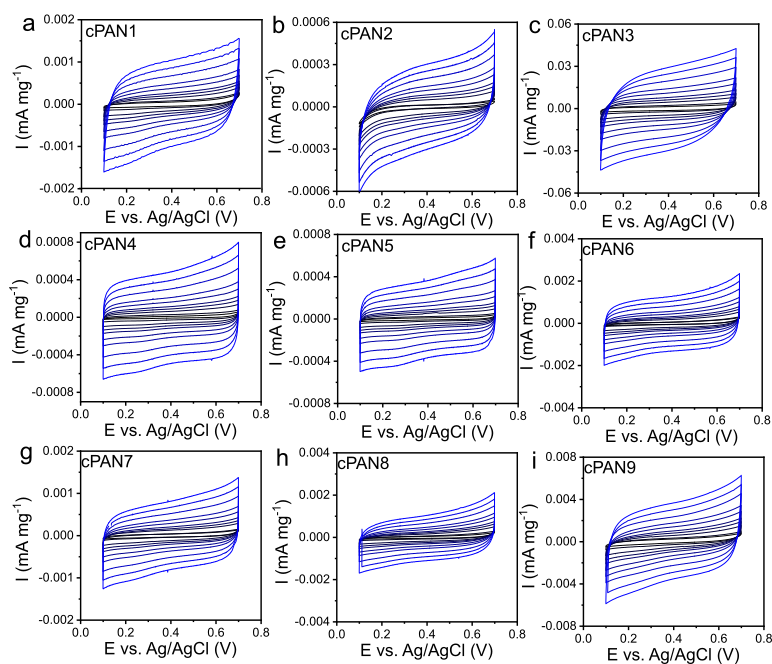


**Fig. S8** Cyclic voltammograms recorded in a non-Faradaic potential range (0.1 to 0.7 V vs. Ag/AgCl) at various scan rates: 10, 20, 50, 75, 100, 150, 200, 300, 400 and 500 mV s⁻¹ for samples cPAN1–cPAN9 in 1 M NaCl aqueous electrolyte


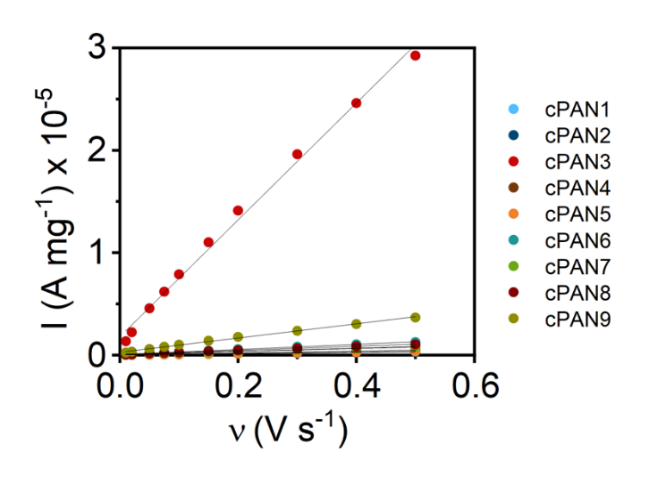


**Fig. S9** Current (measured at 0.4 V vs. Ag/AgCl) as a function of scan rate for samples cPAN1–cPAN9

**Table S8** Calculated double-layer capacitance

| **Sample** | **C_dl_ (μF mg^-1^)** | **R^2^** |
| --- | --- | --- |
| cPAN1 | 1.47 | 0.9965 |
| cPAN2 | 0.59 | 0.9905 |
| cPAN3 | 57.03 | 0.9945 |
| cPAN4 | 0.96 | 0.9985 |
| cPAN5 | 0.71 | 0.9984 |
| cPAN6 | 2.49 | 0.9971 |
| cPAN7 | 1.58 | 0.9964 |
| cPAN8 | 2.02 | 0.9965 |
| cPAN9 | 6.94 | 0.9965 |

**Table S9** Performance comparison of carbon-based electrodes for β-Blocker removal from aquatic matrixes

| **electrode type** | **reactor type** | **geometry** | **treatement time** | **matrix** | **removal efficiencies** | | | **literature** |
| --- | --- | --- | --- | --- | --- | --- | --- | --- |
|  |  |  |  |  | **MET** | **ATE** | **PROP** |  |
| carbon electrodes (carbon nanowall) | flow  reactor | fks^-^ | 120 min | phosphate solution | 81.0% | 69.4% | 94.9% | this study |
|  |  | gyr^+^ |  |  | 33.6% | 23.5% | 91.9% |  |
|  |  | dia^+^ |  |  | 43.1% | 28.9% | 91.2% |  |
|  |  | dia^-^ |  |  | 31.0% | 28.2% | 75.7% |  |
|  |  | pri^-^ |  |  | 43.4% | 42.7% | 65.9% |  |
| graphite-polyvinyl chloride composite | batch  reactor | flat | 60 min | NaCl  electrolyte | >90% | - | - | [S1] |
| Nb/BDD carbon electrode (2k BDD) | batch  reactor | flat | 240 min | medical wastewater | 91.50% | - | - | [S2] |
| Cu-B-Fe functionalized graphite cathode | flow-assisted electro-Fenton reactor | flat | 60 min | hospital wastewater | - | 99.9% | 99.9% | [S3] |
| Boron-doped diamond (BDD) electrodes | batch  reactor | flat | 30 min | Na_2_SO_4_ electrolyte | - | - | 90.4% | [S4] |
| BDD electrode (2.5k NeoCoat) | Batch  reactor | flat | 360 min | Na_2_SO_4_ electrolyte | - | ~80% | - | [S5] |
| Nb/BDD electrode (2.5k ) | flow  filter-press reactor | flat square electrodes | 120 min | Na_2_SO_4_ electrolyte | - | 75 % | - | [S6] |
| BDD electrode (5k NeoCoat) | flow filter-press reactor | flat | 240 min | Na_2_SO_4_ electrolyte | - | ~100 % | - | [S7] |

**Table S10** Degradation products were evaluated using single ion monitoring mode (SIM).

| **Compound name** | **MW [Sg/mol]** | **MW+1**  **[m/z]** | **Refs.** |
| --- | --- | --- | --- |
| **Metoprolol:** | 267.36 | 268.36 | [S8] |
| 4-(2-methoxyethyl)-phenol | 152.19 | 153.19 |  |
| 3-chloro-4-hydroxyphenyl acetic acid | 186.59 | 187.59 |  |
| 1-(propan-2-ylamino) propane-2-ol | 117.19 | 118.19 | [S9]  [S10] |
| 1-(propan-2-ylamino) propane-1,2,3-triol | 151.19 | 152.19 |  |
| Methyl 4-hydroxyphenylacetate | 166.17 | 167.17 |  |
| 2-hydroxy-2(4 hydroxyphenyl) acetic acid | 168.15 | 169.15 |  |
| 4-hydroxybenzaldehyde | 122.12 | 123.12 |  |
| **Propranolol:** | 259.34 | 260.34 | [S11] |
| 1-Naphthol | 144.17 | 145.17 |  |
| 1,4-Naphthoquinone | 158.15 | 159.15 |  |
| 1,7-Dihydronaphto |  |  |  |
| Phthalic anhydride | 148.11 | 149.11 |  |
| Acetamide | 59.07 | 60.07 |  |
| Glycolic acid | 76.05 | 77.05 |  |
| Pyruvic acid | 88.06 | 89.06 |  |
| Malonic acid | 104.06 | 105.06 |  |
|  |  |  |  |
| **Atenolol** | 266 | 267 | [S12] |
| 4-[2-Hydroxy-3-[(1-methylethyl)amino]propoxy]benzamide | 282 | 283 |  |
| 4-[2-Hydroxy-3-amino-propoxy]benzaldehyde | 254 | 255 |  |
| 4-[2-Hydroxy-3-(isopropylamino)propoxy]benzaldehyde | 237 | 238 |  |
| 4-[2-Hydroxy-3-amino-propoxy]benzamide | 224 | 225 |  |
| 4-[3-Aminopropoxy]benzamide | 207 | 207 |  |
|  | 193 | 194 |  |
| 4-(2-Oxopropyl)benzamide | 188 | 189 |  |
| 4-Hydroxybenzamide | 151 | 152 |  |
| 2-Hydroxy-3-(isopropylamino)propionaldehyde | 133 | 134 |  |

**Supplementary References**

1. Z.H. Mussa, F.F. Al-Qaim, A. Yuzir, K. Shameli, Electrochemical removal of metoprolol using graphite-polyvinyl chloride composite as anode. IOP Conf. Ser. Earth Environ. Sci. **479**(1), 012022 (2020). <https://doi.org/10.1088/1755-1315/479/1/012022>
2. E. Bączkowska, M. Pierpaoli, F. Gamoń, A. Luczkiewicz, S. Fudala-Ksiazek et al., On-site medical wastewater treatment enabling sustainable water reclamation: Merged advanced oxidation process for disinfection, toxicity, and contaminants removal. J. Water Process. Eng. **72**, 107562 (2025). <https://doi.org/10.1016/j.jwpe.2025.107562>
3. H. Nsubuga, C. Basheer, M. Baseer Haider, An enhanced beta-blockers degradation method using copper-boron-ferrite supported graphite electrodes and continuous droplet flow-assisted electro-Fenton reactor. Sep. Purif. Technol. **221**, 408–420 (2019). <https://doi.org/10.1016/j.seppur.2019.03.095>
4. H.A. Nájera-Aguilar, R. Mayorga-Santis, R.F. Gutiérrez-Hernández, A. Santiesteban-Hernández, F.J. Rodríguez-Valadez et al., Propranolol degradation through processes based on the generation of hydroxyl free radical. J. Water Health **20**(1), 216–226 (2022). <https://doi.org/10.2166/wh.2021.156>
5. A. Balseviciute, I. Patiño-Cantero, J. Carrillo-Abad, J.J. Giner-Sanz, M. García-Gabaldón et al., Degradation of multicomponent pharmaceutical mixtures by electrochemical oxidation: Insights about the process evolution at varying applied currents and concentrations of organics and supporting electrolyte. Sep. Purif. Technol. **362**, 131697 (2025). <https://doi.org/10.1016/j.seppur.2025.131697>
6. A.N. Arenhart Heberle, M. García-Gabaldón, E.M. Ortega, A.M. Bernardes, V. Pérez-Herranz, Study of the atenolol degradation using a Nb/BDD electrode in a filter-press reactor. Chemosphere **236**, 124318 (2019). <https://doi.org/10.1016/j.chemosphere.2019.07.049>
7. S.W. da Silva, J.M. do Prado, A.N.A. Heberle, D.E. Schneider, M.A.S. Rodrigues et al., Electrochemical advanced oxidation of Atenolol at Nb/BDD thin film anode. J. Electroanal. Chem. **844**, 27–33 (2019). <https://doi.org/10.1016/j.jelechem.2019.05.011>
8. M.E.H. Bergmann, K. Kresse, J. Rollin, J. Hartmann, Metoprolol behaviour in drinking water electrolysis using mixed oxide and boron doped diamond anodes. Water Pract. Technol. **5**(2), wpt2010032 (2010). <https://doi.org/10.2166/wpt.2010.032>
9. H. Olvera-Vargas, T. Cocerva, N. Oturan, D. Buisson, M.A. Oturan, Bioelectro-Fenton: a sustainable integrated process for removal of organic pollutants from water: application to mineralization of metoprolol. J. Hazard. Mater. **319**, 13–23 (2016). <https://doi.org/10.1016/j.jhazmat.2015.12.010>
10. J. Radjenovic, B.I. Escher, K. Rabaey, Electrochemical degradation of the β-blocker metoprolol by Ti/Ru 0.7 Ir_0.3_ O_2_ and Ti/SnO 2-Sb electrodes. Water Res. **45**(10), 3205–3214 (2011). <https://doi.org/10.1016/j.watres.2011.03.040>
11. E. Isarain-Chávez, P.L. Cabot, F. Centellas, R.M. Rodríguez, C. Arias et al., Electro-Fenton and photoelectro-Fenton degradations of the drug beta-blocker propranolol using a Pt anode: Identification and evolution of oxidation products. J. Hazard. Mater. **185**(2–3), 1228–1235 (2011). <https://doi.org/10.1016/j.jhazmat.2010.10.035>
12. K. Govindan, V.D.W. Sumanasekara, A. Jang, Mechanisms for degradation and transformation of β-blocker atenolol *via* electrocoagulation, electro-Fenton, and electro-Fenton-like processes. Environ. Sci. Water Res. Technol. **6**(5), 1465–1481 (2020). <https://doi.org/10.1039/D0EW00114G>
